# Pre‐ADMET studies of 5‐(3′,4′‐dihydroxyphenyl)‐γ‐valerolactone, the bioactive intestinal metabolite of proanthocyanidins

**DOI:** 10.1002/ardp.202400575

**Published:** 2024-11-11

**Authors:** Larissa Della Vedova, Islam Husain, Yan‐Hong Wang, Hari Babu Kothapalli, Francesca Gado, Giovanna Baron, Simone Manzi, Paolo Morazzoni, Giancarlo Aldini, Ikhals A. Khan

**Affiliations:** ^1^ Department of Pharmaceutical Sciences University of Milan Milan Italy; ^2^ National Center for Natural Products Research, School of Pharmacy The University of Mississippi Oxford Mississippi USA; ^3^ Divisione Nutraceutica Distillerie Umberto Bonollo S.p.A Mestrino Italy

**Keywords:** 5‐(3′,4′‐dihydroxyphenyl)‐γ‐valerolactone, HDI, pre‐ADMET

## Abstract

5‐(3′,4′‐Dihydroxyphenyl)‐γ‐valerolactone (VL) is a bioactive metabolite resulting from the gut microbial metabolism of proanthocyanidins and flavonoids, known for its health‐promoting effects, including antidiabetic and anti‐inflammatory activities. Although VL has been observed in different in vivo studies, its pre‐absorption, distribution, metabolism, excretion, toxicity (ADMET) properties have rarely been investigated. This study aims to address this gap by evaluating the pre‐ADMET properties of VL for the first time. Also, the understanding of these properties is significant for correlating the encountered activities to this metabolite. In vitro absorption studies revealed that VL is rapidly metabolized and absorbed as its sulfate phase II conjugate (valerolactone sulfate), which enters systemic circulation and mildly activates the Breast Cancer Resistance Protein efflux transporter. In human S9 liver fraction, a mixture of liver enzymes used to simulate in vivo liver metabolism, VL is metabolized into glucuronic phase II conjugates (valerolactone glucuronide 1 [VLG1] and 2 [VLG2]) with a half‐life of 8.72 min and an 80% conversion rate. In human liver microsomes, VL is metabolized at a slower rate (half‐life of 23.08 min), suggesting that oxidative metabolism is secondary. Additionally, VL did not activate the pregnane X receptor or inhibit Cytochrome P3A4 (CYP3A4) and Cytochrome P1A2 (CYP1A2) enzymes, indicating no risk of herb–drug interactions with coadministered prescription drugs.

## Graphical abstract

The pre‐ADMET properties of 5‐(3′,4′‐dihydroxyphenyl)‐γ‐valerolactone (VL) are evaluated for the first time. VL is rapidly metabolized and absorbed as its sulfate phase II conjugate (valerolactone sulfate). In the human S9 liver fraction, VL is metabolized into glucuronic phase II conjugates. In human liver microsomes, it is metabolized at a slower rate, suggesting that oxidative metabolism is secondary.

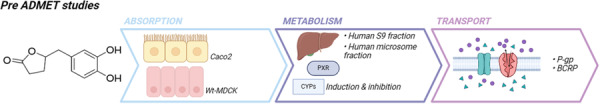

## 1 INTRODUCTION

**Figure 1:**
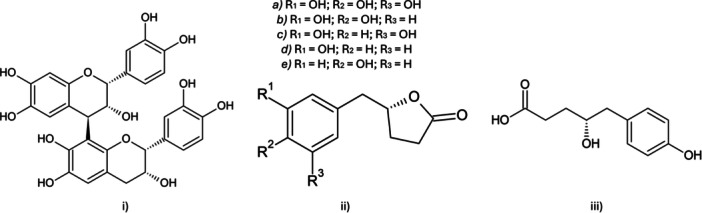
Chemical structures of (i) a representative PAC (i.e., procyanidin B2); (ii) PVLs: (a) 5‐(3′,4′,5′‐trihydroxyphenyl)‐γ‐valerolactone, (b) 5‐(3′,4′‐dihydroxyphenyl)‐γ‐valerolactone, (c) 5‐(3′,5′‐dihydroxyphenyl)‐γ‐valerolactone, (d) 5‐(3′‐hydroxyphenyl)‐γ‐valerolactone, and (e) 5‐(4′‐hydroxyphenyl)‐γ‐valerolactone; (iii) phenyl 4‐OH valeric acid. PAC, proanthocyanidin; PVL, hydroxy‐phenyl‐γ‐valerolactone.

Proanthocyanidins (PACs) and flavan‐3‐ols are natural compounds derived from flavonoids belonging to the family of polyphenols and are present in the flowers, nuts, fruits, bark, and seeds of various plants, as a defense against biotic and abiotic stressors. Chemically, PACs are highly hydroxylated structures built from flavan‐3‐ol blocks, forming oligomeric structures of at least two units.^[^ ^1^, ^2^ ^]^ Flavonoids are associated with a broad spectrum of health‐promoting effects and are an indispensable component in a variety of nutraceutical, pharmaceutical, medicinal, and cosmetic applications.^[^ ^2^ ^]^ More specifically, PACs have been extensively reported in the literature for their systemic anti‐inflammatory, antiobesity, neuroprotectant, and anticancer activities, but also for their local benefits in the gastro‐intestinal (GI) tract, which is considered their main target organ. Specifically, PACs modulate hormone secretions, epithelial transport, and GI transit and they display local anti‐inflammatory and antioxidant activities, which favor ulcer healing and mucosa integrity leading to a reduced risk of developing colorectal cancer.^[^ ^3^, ^4^, ^5^, ^6^ ^]^ Besides their pharmacological activities, PACs are well known for the complexity of their structure and their large degree of polymerization, limiting the investigation of their bioavailability and metabolism and therefore the explanation of their encountered bioactivities.^[^ ^7^ ^]^ As a consequence of the increasing evidence of their health‐promoting effects, PAC application in the food and pharmaceutical industry could be very promising, but this faces obstacles and limitations, due to their encountered bioavailability issues.^[^ ^8^ ^]^ Also, the concentrations they reach in vivo in the circulatory system are lower than would be expected given the health benefits they display. Indeed, only monomers and dimeric PACs can be absorbed in the GI tract, oligomeric and polymeric PACs are not bioavailable and are found as intact parent compounds throughout the GI tract and in feces.^[^ ^8^ ^]^ Most ingested PACs reach the colon and are subjected to fermentation by gut microbiota in hydroxy‐phenyl‐γ‐valerolactones (PVLs) and their related phenyl‐4‐hydroxyvaleric acids, which can be subsequently degraded into low molecular weight phenolic acids and aromatic compounds (Figure 1).^[^ ^9^, ^10^ ^]^

**Figure 2:**
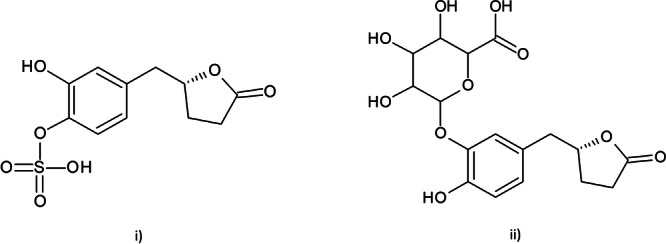
Chemical structures of (i) 3ʹ‐OH‐PVL‐4ʹ‐sulfate and (ii) 4ʹ‐OH‐PVL‐3ʹ‐glucuronide. PVL, hydroxy‐phenyl‐γ‐valerolactone.

These small phenolics can be absorbed and in turn subjected to phase II metabolism at the hepatocyte level to produce conjugated metabolites (such as sulfate, glucuronide, and methoxy) that can circulate through the system and then be excreted in urine.^[^ ^11^ ^]^ These compounds are therefore potentially available to target tissues and organs influencing (patho)physiological scenarios. For these reasons, interest in these phytochemicals has grown, especially concerning their possible metabolism mediated by the gut microbiota; in fact, these studies have highlighted the importance of PVLs, which are nowadays established as the main microbial metabolites of PACs. These PVL metabolites have been extensively studied in terms of their nomenclature, formation, synthetic steps for preparation of their standards and analytical methods for their identification. Among them, 5‐(3′,4′‐dihydroxyphenyl)‐γ‐valerolactone (VL) has been found to be the most abundant, formed by the gut microbiota after flavonoid‐rich food and supplement intake. In recent years, interest in this metabolite has increased, particularly investigation of its formation in the intestine, its renal excretion, and its biological activities. Parmenter et al. in 2023 reported the results of a five‐way randomized crossover trial and of an observational cross‐sectional study quantifying 10 urinary PVLs using liquid chromatography‐tandem mass spectrometry (MS) following a dietary flavan‐3‐ol intake.^[^ ^12^ ^]^ The two major urinary metabolites identified are secondary conjugated metabolites of VL, 3ʹ‐OH‐PVL‐4ʹ‐sulfate, and 4ʹ‐OH‐PVL‐3ʹ‐glucuronide (Figure 2) and are subsequently recommended as biomarkers for dietary flavan‐3‐ol exposure since they reflect flavan‐3‐ol intake in a dose‐dependent way.^[^ ^12^ ^]^

A recent study by Baron et al. focuses on an integrated in vitro and in vivo approach followed by a biological and proteomic analysis of a highly standardized grape seed extract prepared by Distillerie Bonollo Umberto S.p.A (commercial name Ecovitis) highlighting VL as being mainly responsible for the beneficial effects of PACs at the systemic level, more specifically of the anti‐inflammatory and antioxidant effects usually related to PAC assumption. The antioxidant effect was demonstrated to be displayed through nuclear factor E2‐related factor 2 (Nrf2) activation thanks to the presence of a catechol moiety in VL which is essential for the oxidative conversion to *ortho*‐quinone which binds the Cysteine of Kelch‐like ECH‐associated protein 1 (Keap 1) activating the Nrf2 pathway able to inhibit also the inflammatory NF‐κB signaling pathway. Consequently, the two VL metabolites 3ʹ‐OH‐PVL‐4ʹ‐sulfate and 4ʹ‐OH‐PVL‐3ʹ‐glucuronide, also identified by Baron et al., are basically circulating inactive forms subjected to enzymatic conversion to the active form which explains the systemic activity of a polyphenol‐rich supplement.^[^ ^13^ ^]^ Although over the last few years much progress has been made in the understanding of this process, more effort and studies are needed to fully clarify the mechanisms of action and absorption and the respective effects of VL. Furthermore, PACs, flavan‐3‐ols, and flavonoids in general have recently come to be seen not only as food but also as potential nutraceuticals, so it is important to investigate possible herb–drug interactions (HDI) in the case of flavonoid‐enriched supplements concomitantly taken with prescription drugs.

In this scenario, VL is the main bioactive intestinal metabolite after PACs and polyphenol intake but so far pre‐absorption, distribution, metabolism, excretion, toxicity (ADMET) properties have not been investigated.^[^ ^14^ ^]^ For these reasons, our work aims to understand and demonstrate the mechanism of absorption and efflux and the metabolic stability, cytotoxicity, and drug‐like properties of VL, to more fully understand the correlation between PACs and VL biological activities.^[^ ^15^ ^]^ Moreover, a better understanding of pre‐ADMET properties of VL can guide us to the identification of a possible new LEAD compound that can represent a new scaffold for the synthesis of innovative compounds with marked in vivo antioxidant and anti‐inflammatory activities.

## 2 RESULTS AND DISCUSSION

### 2.1 Cytotoxicity assay

VL (1.5–50 µM) had no significant effect on the viability of LS174T, HepG2, and Caco2 cells up to 24 and 48 h. Further, 6 h exposure of VL, the same concentration range, had no untoward effects on wild type Madin‐Darby canine kidney (Wt‐MDCK) and Caco2 cell growth, and >90% of cells remained viable. In contrast, exposure to 20 μM doxorubicin and camptothecin dramatically reduced the viability of all tested cell lines under the same experimental conditions (data not shown).

### 2.2 Permeability assay

**Figure 3:**
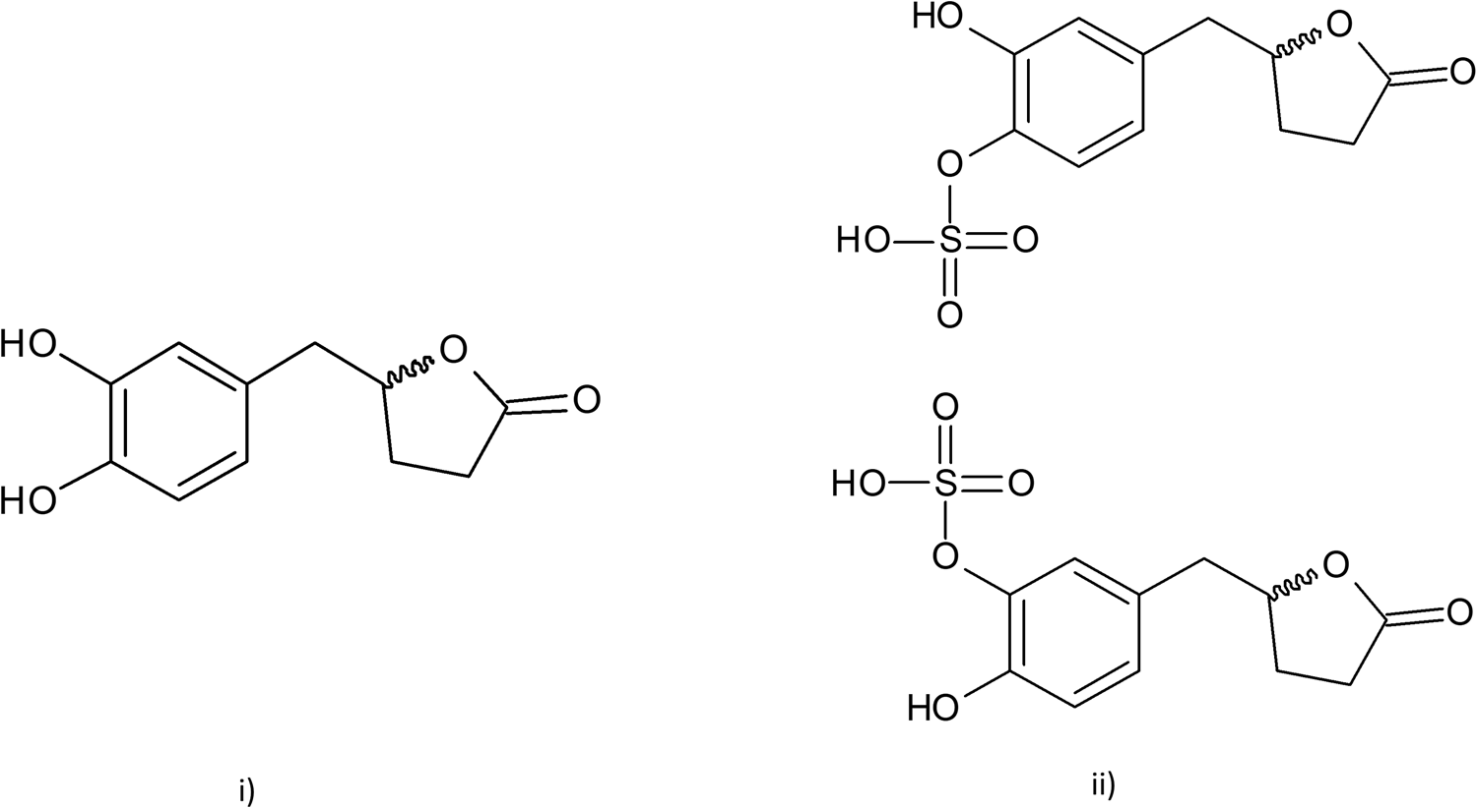
Chemical structures of (i) VL used in this study and (ii) the metabolite VLS, where the sulfate group might be conjugated or in position 4′ or in position 3′. VL, 5‐(3′,4′‐dihydroxyphenyl)‐γ‐valerolactone; VLS, valerolactone sulfate.

**Figure 4:**
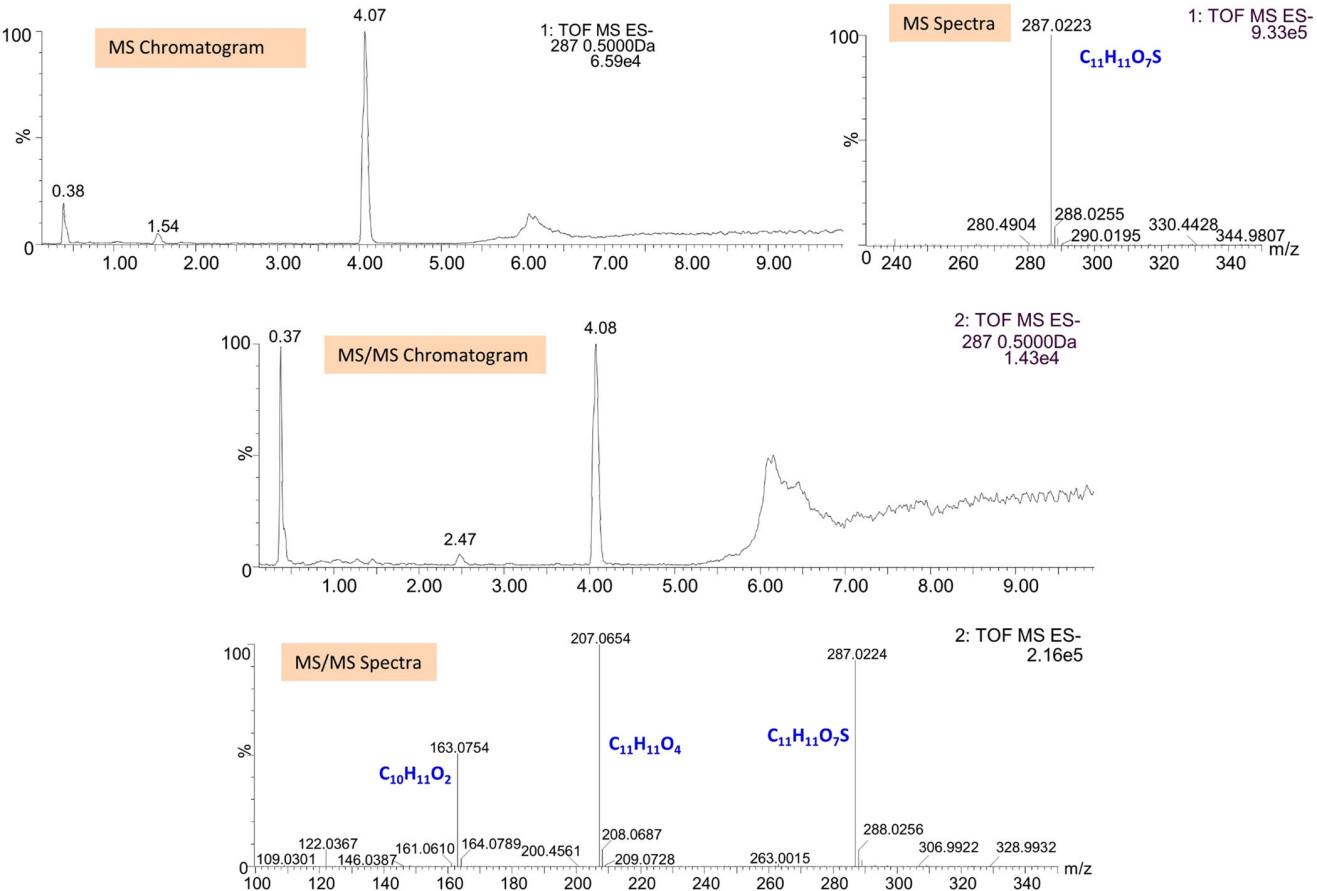
VLS LC‐HRMS chromatograms and spectra. ES, electrospray; LC‐HRMS, liquid chromatography with high‐resolution mass spectrometer; MS, mass spectrometry; TOF, time‐of‐flight; VLS, valerolactone sulfate.

**Figure 5:**
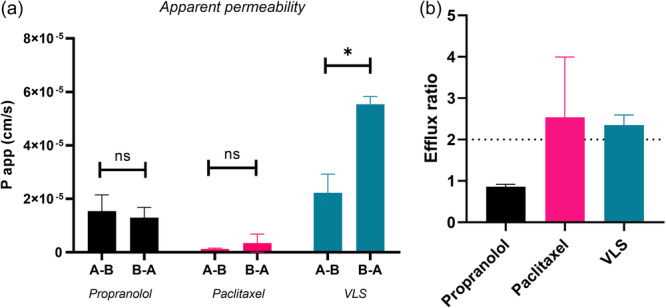
(Panel A) The apparent permeability of controls (propranolol and paclitaxel) and of the metabolite VLS: VLS shows a higher excretion than absorption. (Panel B) The calculated efflux ratios: paclitaxel and VLS are excreted preferentially. Results are relative to the Caco2 cell model. Data are reported as mean ± SD (*N* = 4). The statistical significance difference was analyzed by unpaired *t* test analysis with a 95% confidence interval. *p > 0.05; ns, not significant. VLS, valerolactone sulfate.

In vitro intestinal permeability of VL was evaluated by the application of two different in vitro models, the 21‐day Caco2 and 5‐day Wt‐MDCK. Apparent permeability (*P*_app_) values are commonly used to predict the rate of intestinal absorption of a compound. Compounds with *P*_app_ value greater than 1 × 10^−6^ cm/s are considered to have good intestinal absorption; incomplete absorption occurs if the values are close to 1 × 10^−7^ cm/s.^[^ ^16^ ^]^ Results are shown in Figure 5 and Table 1. VL is rapidly metabolized into its sulfate metabolite, valerolactone sulfate (VLS), which in the Caco2 model showed a *P*_app_ value of 2.225 × 10^−5^ cm/s in the absorptive direction (*A*–*B*) and 5.535 × 10^−5^ cm/s in the secretory direction (*B*–*A*). The formation rate of VLS is calculated as the slope of the line obtained by linear regression, interpolating time with VLS concentrations in the linear formation phase. In the *A*–*B* experiment, the slope of the line passing through the origin is 5.64 × 10^−^^5 ^M/s, while in the *B*–*A* experiment it is 8.11 × 10^−^^5 ^M/s. In both cases, the formation rates are of the same order of magnitude, indicating that the formation rate does not depend on the experiment's direction. Additionally, this rate indicates rapid formation, absorption, and transport of VLS at the intestinal level. VLS was eluted at 4.08 min with molecular ions at *m/z* 287.0227 ([M–H]^−^, calc. 287.0225). The key fragments showed at *m/z* 207.0654 ([M–H–SO_3_]^−^) and 163.0754 ([M–H–SO_3_–CO_2_]^−^). Figure 3 shows the chemical structures of VL and VLS, while Figure 4 shows the chromatogram and MS spectra of VLS.

**Table 1:** Experimental mean and calculated standard deviation of the apparent permeability and efflux ratio of each tested compound.

Compound name	*P*_app_ *A*–*B* (mean ± SD) (cm/s)	*P*_app_ *B*–*A* (mean ± SD) (cm/s)	Efflux ratio (mean ± SD)
Propranolol	1.539 × 10^−5^ ± 6.053 × 10^−6^	1.296 × 10^−5^ ± 3.884 × 10^−6^	0.859 ± 0.085
Paclitaxel	1.202 × 10^−6^ ± 3.607 × 10^−7^	3.421 × 10^−6^ ± 3.392 × 10^−6^	2.537 ± 2.061
VLS	2.225 × 10^−5^ ± 7.000 × 10^−6^	5.535 × 10^−5^ ± 2.899 × 10^−6^	2.351 ± 0.341

*Note*: Data are reported as mean ± SD (*N* = 4). Results are relative to the Caco2 cell model.

Abbreviation: VLS, valerolactone sulfate.

The calculated apparent permeability is considered acceptable for the oral route of administration. The efflux ratio of 2.351 suggests that VLS may be a substrate of an efflux transporter. Commonly efflux ratio parameter is used in in vitro studies to consider the possible involvement of *P*‐glycoprotein (P‐gp), but the main determinant for the efflux is the affinity of the tested compound to the efflux transporter and the rate of passive transport of the drug. Specifically, when passive transport is high, the amount of drug secreted by the efflux transporter can be rapidly reabsorbed, further investigation is needed to clarify the VLS uptake.^[^ ^17^ ^]^ Also, due to the lack in this study of a VLS standard, to calculate a relative amount of the metabolite formed over time, the VL standard curve was used for correlating the peak area of the multiple reaction monitoring (MRM) *m/z* 207.1–162.9 transition with the VL concentration. Therefore, VLS concentrations are relative and expressed in nM of VL. The highly absorbed drug propranolol (positive control) showed apparent permeability values of 1.539 × 10^−5^ cm/s in the *A*–*B* direction and 1.296 × 10^−5^ cm/s in the *B*–*A* direction in the Caco2 model, and the results obtained are in line with previous data reported in the literature.^[^ ^18^ ^]^ Likewise, the poorly absorbed drug paclitaxel (negative control) showed apparent permeability values of 1.202 × 10^−6^ cm/s in the *A*–*B* direction and 3.421 × 10^−6^ cm/s in the *B*–*A* direction in the Caco2 model, as previously reported in the literature, demonstrating the reproducibility and accuracy of the in vitro model used.^[^ ^19^ ^]^ Caco2 cell model results are shown in Figure 5 and Table 1.

**Figure 6:**
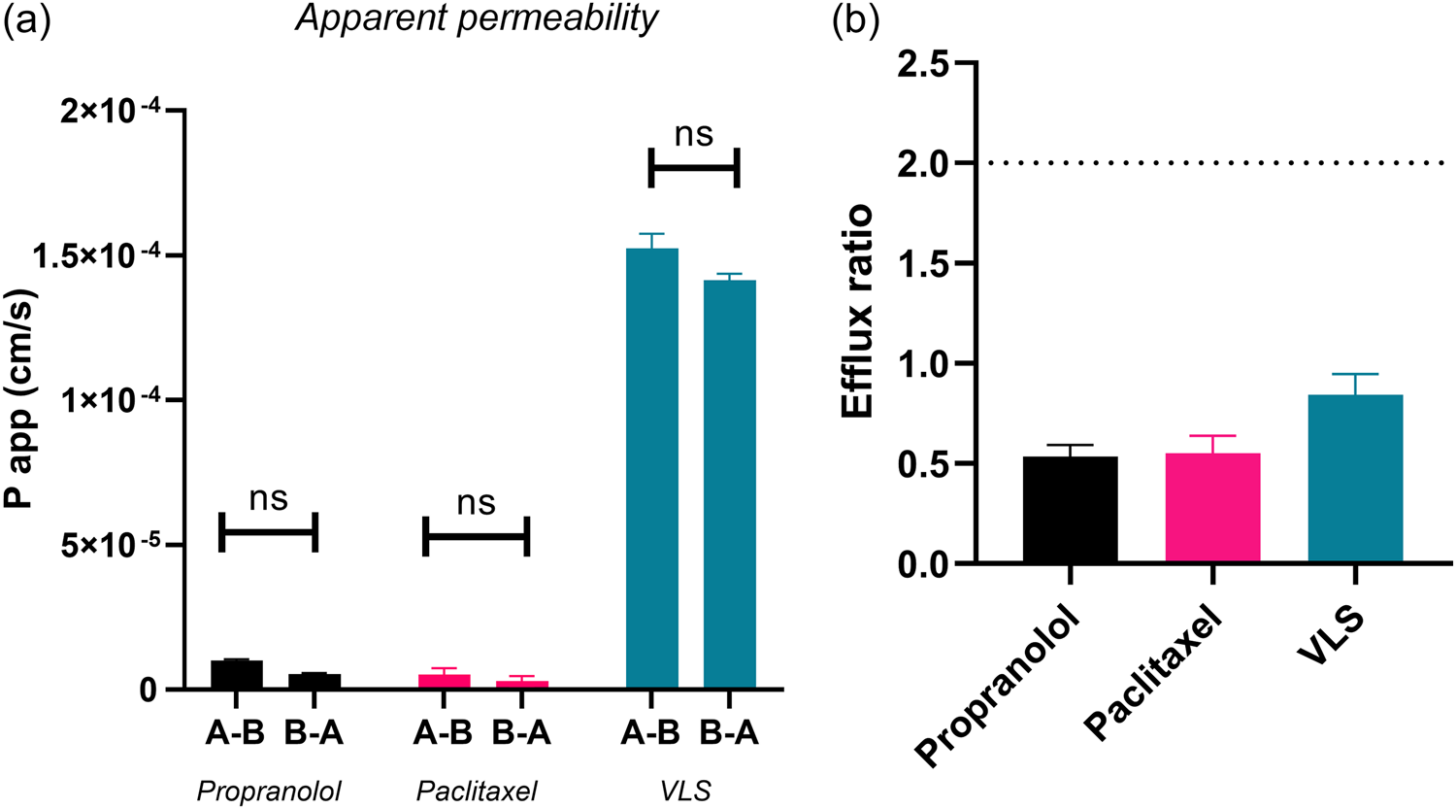
(Panel A) The apparent permeability of controls (propranolol and paclitaxel) and of the metabolite VLS: all tested compounds do not show a preferential transport direction. (Panel B) The calculated efflux ratios: all tested compounds are not excreted. Results are relative to the Wt‐MDCK cell model. Data are reported as mean ± SD (*N* = 4). The statistical significance difference was analyzed by unpaired *t* test analysis with a 95% confidence interval. ns, not significant; VLS, valerolactone sulfate; Wt‐MDCK, wild type Madin‐Darby canine kidney.

In the Wt‐MDCK model VL is rapidly metabolized in VLS and its *P*_app_ value is 1.733 × 10^−5^ cm/s in the absorptive direction (*A*–*B*) and 1.443 × 10^−5^ cm/s in the secretory direction (*B*–*A*). The efflux ratio of 0.843 shows that VLS in this cellular model is not a substrate of efflux transporters and additionally its calculated apparent permeability is considered acceptable for the oral route of administration. Apparent permeability values of propranolol (1.005 × 10^−5^ cm/s in the *A*–*B* direction and 5.364 × 10^−6^ cm/s in the *B*–*A* direction) and paclitaxel (5.183 × 10^−6^ cm/s in the *A*–*B* direction and 2.952 × 10^−6^ cm/s in the *B*–*A* direction) are comparable with *P*_app_ values calculated using the Caco2 model, showing that the Wt‐MDCK cellular model is a robust and accurate in vitro model. Results are shown in Figure 6 and Table 2.

**Table 2:** Experimental mean and the calculated standard deviation of the apparent permeability and efflux ratio of each tested compound.

Compound name	*P*_app_ *A*–*B* (mean ± SD) (cm/s)	*P*_app_ *B*–*A* (mean ± SD) (cm/s)	Efflux ratio (mean ± SD)
Propranolol	1.005 × 10^−5^ ± 4.586 × 10^−7^	5.364 × 10^−6^ ± 3.377 × 10^−7^	0.535 ± 0.058
Paclitaxel	5.183 × 10^−6^ ± 2.269 × 10^−6^	2.952 × 10^−6^ ± 1.708 × 10^−6^	0.550 ± 0.089
VLS	1.733 × 10^−4^ ± 2.500 × 10^−5^	1.443 × 10^−4^ ± 4.193 × 10^−6^	0.843 ± 0.105

*Note*: Data are reported as mean ± SD (*N* = 4). Results are relative to the Wt‐MDCK cell model.

Abbreviation: VLS, valerolactone sulfate.

In addition, at the end of the transport experiments, the monolayer integrity was confirmed by measuring the trans‐epithelial electrical resistance (TEER) across the monolayer, which was higher than 600 Ω/cm^2^ for the Caco2 model and higher than 90 Ω/cm^2^ for the Wt‐MDCK model, after blank subtraction.

### 2.3 Efflux transporter modulation

As mentioned in Section 2.2, to more fully understand the role of efflux transporters in VLS uptake, the transport of VL was first monitored in the presence of inhibitors of P‐gp (cyclosporine A [CsA]) and Breast Cancer Resistance Protein (BCRP) (elacridar)^[^ ^20^ ^]^ and then with an inductor of P‐gp (rifampicin [RIF]), performing the Rh‐123 uptake‐based efflux assay in the differentiated Caco2 cell line. Considering the results just discussed and the rate of VL metabolization, in both the inhibition and induction experiments the substrate of P‐gp and BCRP is the intestinal phase II metabolite VLS, and its concentration can be assumed to be the same as the initial concentration of tested VL.

#### 2.3.1 Inhibition

**Figure 7:**
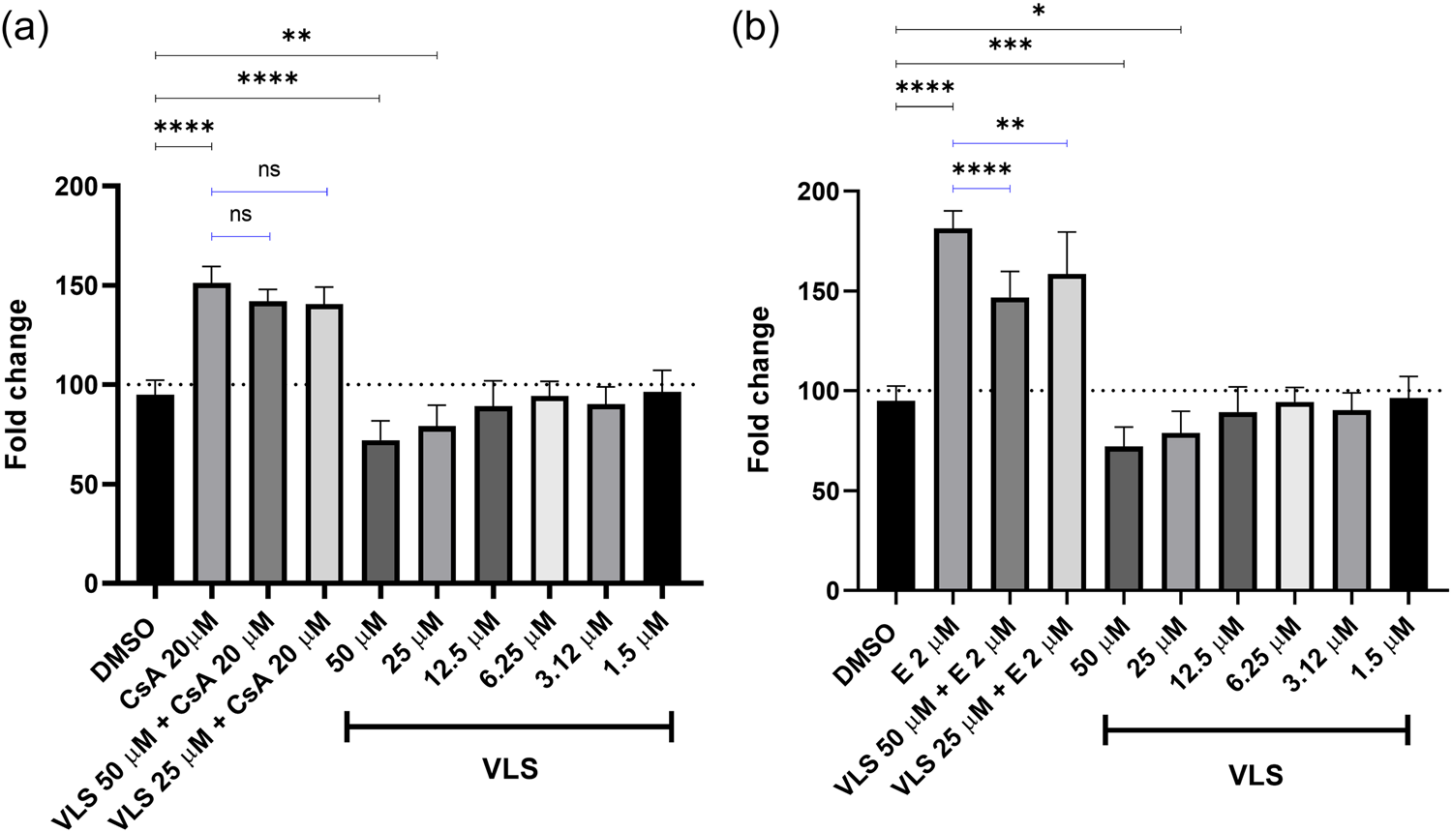
Efflux transporter inhibition assay results: (panel A) shows that when VLS is tested with ciclosporin A, no significant results in Rh‐123 accumulation are observed; (panel B) shows that when VLS is tested with elacridar, a significant reduction of accumulation of Rh‐123 is observed with a dose‐dependent effect. Data are reported as mean ± SD (*N* = 3). The statistical significance difference was analyzed by one‐way analysis of variance followed by Bonferroni's multiple comparisons test with a 95% confidence interval. *p > 0.05; **p < 0.01; ***p < 0.005; ****p < 0.0001; ns, not significant. CsA, cyclosporine A; DMSO, dimethyl sulfoxide; E, elacridar; VLS, valerolactone sulfate.

CsA is a well‐known first‐generation P‐gp inhibitor^[^ ^21^ ^]^ and when incubated for 90 min at the concentration of 20 µM it inhibits the mature P‐gp transporter, with an intracellular accumulation of Rh‐123 1.5‐fold higher than the control. VLS in a higher concentration range (50–12.5 µM) shows a reduced intracellular accumulation of Rh‐123 (72%–89%), lower than the 0.1% dimethyl sulfoxide (DMSO) vehicle control (100%). This result reveals a potential slight activation of an efflux pump. For this reason, the Rh‐123 uptake was evaluated treating cells for 90 min with both CsA 20 µM and VLS, and with both elacridar 2 µM and VLS (VL initial concentration of 50 and 25 µM). When VLS is tested in cotreatment with CsA, nonsignificant results are observed, meaning that VLS does not affect P‐gp pump activation. Elacridar (E) is a potent orally active P‐gp and BCRP inhibitor, as reported in the literature,^[^ ^20^ ^]^ and when VLS is tested in cotreatment with elacridar a significant reduction (from 1.8‐ to 1.4‐folds of the control) of intracellular accumulation of Rh‐123 is observed with a dose‐dependent effect as the concentration of VLS increases, hence VLS is a BCRP substrate and at higher doses VLS slightly activates the BCRP efflux transporter. Results are reported in Figure 7.

#### 2.3.2 Induction

**Figure 8:**
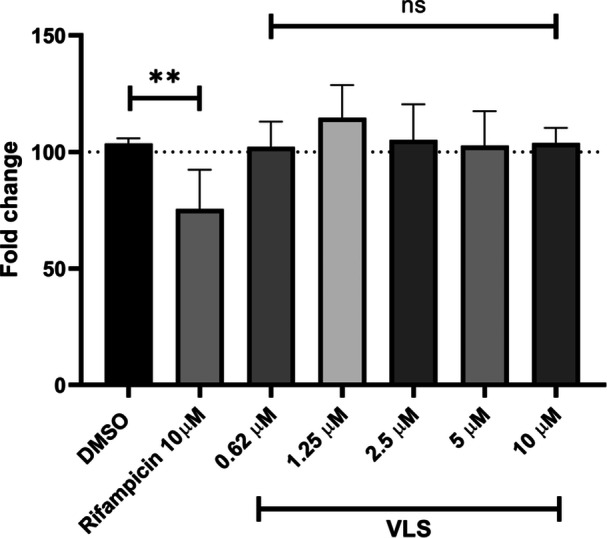
As shown, VLS has no significant effect on P‐gp induction after 48 h of treatment. Data are reported as mean ± SD (*N* = 3). The statistical significance difference was analyzed by one‐way analysis of variance followed by Bonferroni's multiple comparisons test with a 95% confidence interval. **p < 0.01; ns, not significant. DMSO, dimethyl sulfoxide; P‐gp, *P*‐glycoprotein; VLS, valerolactone sulfate.

RIF is a widely known molecule with P‐gp induction activity,^[^ ^22^ ^]^ and it was used as a positive control (final concentration 10 µM) after 48 h treatment reducing the intracellular accumulation of Rh‐123 to 75% with respect to 0.1% DMSO vehicle control (100%). VLS was evaluated in the concentration range from 2.5 to 10 µM, and no significant data of P‐gp induction are shown, as reported in Figure 8. Hence, the rapidly formed intestinal metabolite VLS has no effect on P‐pg downstreaming, reducing any risk of a lower therapeutic effect in the case of herb and drug coadministration.^[^ ^23^ ^]^

### 2.4 In vitro metabolism

**Figure 9:**
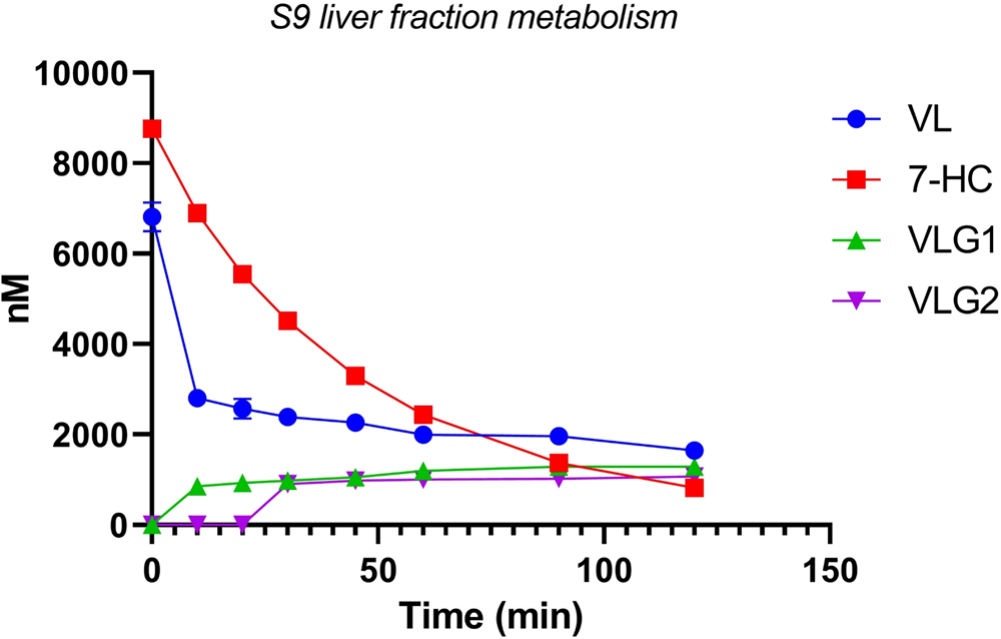
S9 liver fraction metabolism of VL: VL is rapidly metabolized into its glucuronic conjugates VLG1 and VLG2 (*t*_½_ 8.72 min). 7‐Hydroxy coumarin (7‐HC) (positive control). Data are reported as mean ± SD (*N* = 4). Some error bars are not shown on the graph due to their negligible size. VL, 5‐(3′,4′‐dihydroxyphenyl)‐γ‐valerolactone; VLG, valerolactone glucuronide.

**Figure 10:**
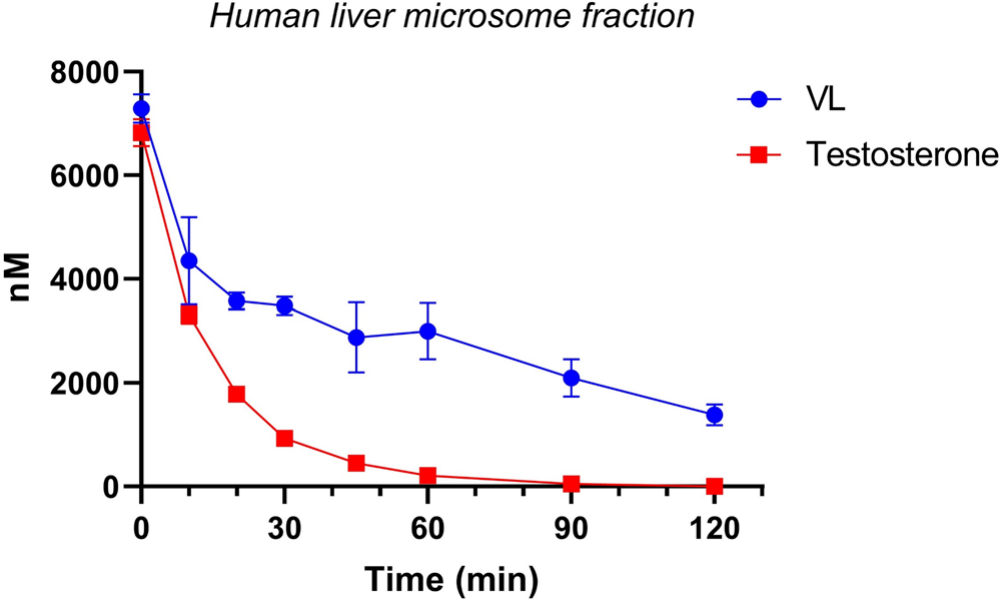
Human liver microsomes S9 fraction metabolism of VL: VL has a slower metabolization rate (*t*_½_ 23.08 min), hence is less subject to phase I metabolism. Testosterone is the positive control. Data are reported as mean ± SD (*N* = 4). Some error bars are not shown on the graph due to their negligible size. VL, 5‐(3′,4′‐dihydroxyphenyl)‐γ‐valerolactone.

Phases I and II metabolisms of VL are evaluated using the human liver microsome (HLM) and liver S9 fraction, a mixture of liver enzymes used to simulate in vivo liver metabolism, and by performing the metabolism assay as reported in Section 5.5. Liver metabolism is very important as it determines the systemic bioavailability of a compound. Phase I reactions consist of the addition or unmasking of functional polar moieties by oxidation (CYP450 or FMO) or hydrolysis (esterases), whereas phase II reactions consist of the conjugation of the compound with small, endogenous substances carried out by the uridine diphosphate (UDP)‐glucuronosyltransferases (UGT). The assessment of phases I and II metabolism was carried out in HLM (phase I) and S9 fractions (phases I and II). Prediction of in vivo drug clearance can be made from the in vitro intrinsic clearance (CLint') data of a drug,^[^ ^24^ ^]^ whereas the measurement of in vitro microsomal half‐life (*t*_½_) is the simplest approach for determining CLint' (mL/min/kg).^[^ ^23^ ^]^ VL is stable under negative control experimental conditions (data not shown). VL is found to be rapidly degraded in the S9 fraction, with a half‐life *t*_½_ of 8.72 min and an intrinsic clearance of 0.784 µM/min (Figure 9), but degraded at a slower rate in HLM with a half‐life *t*_½_ of 23.08 min and an intrinsic clearance of 0.300 µM/min (Figure 10).

**Figure 11:**
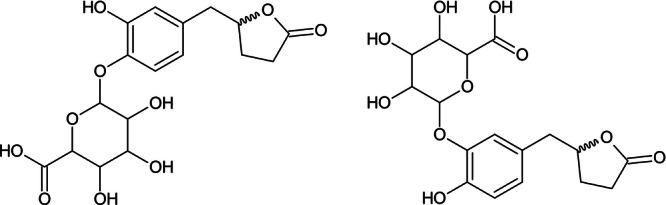
Chemical structures of VLG1 and VLG2; by LC‐HRMS these two different phase II metabolites are not distinguishable. LC‐HRMS, liquid chromatography with high‐resolution mass spectrometer; VLG, valerolactone glucuronide.

**Figure 12:**
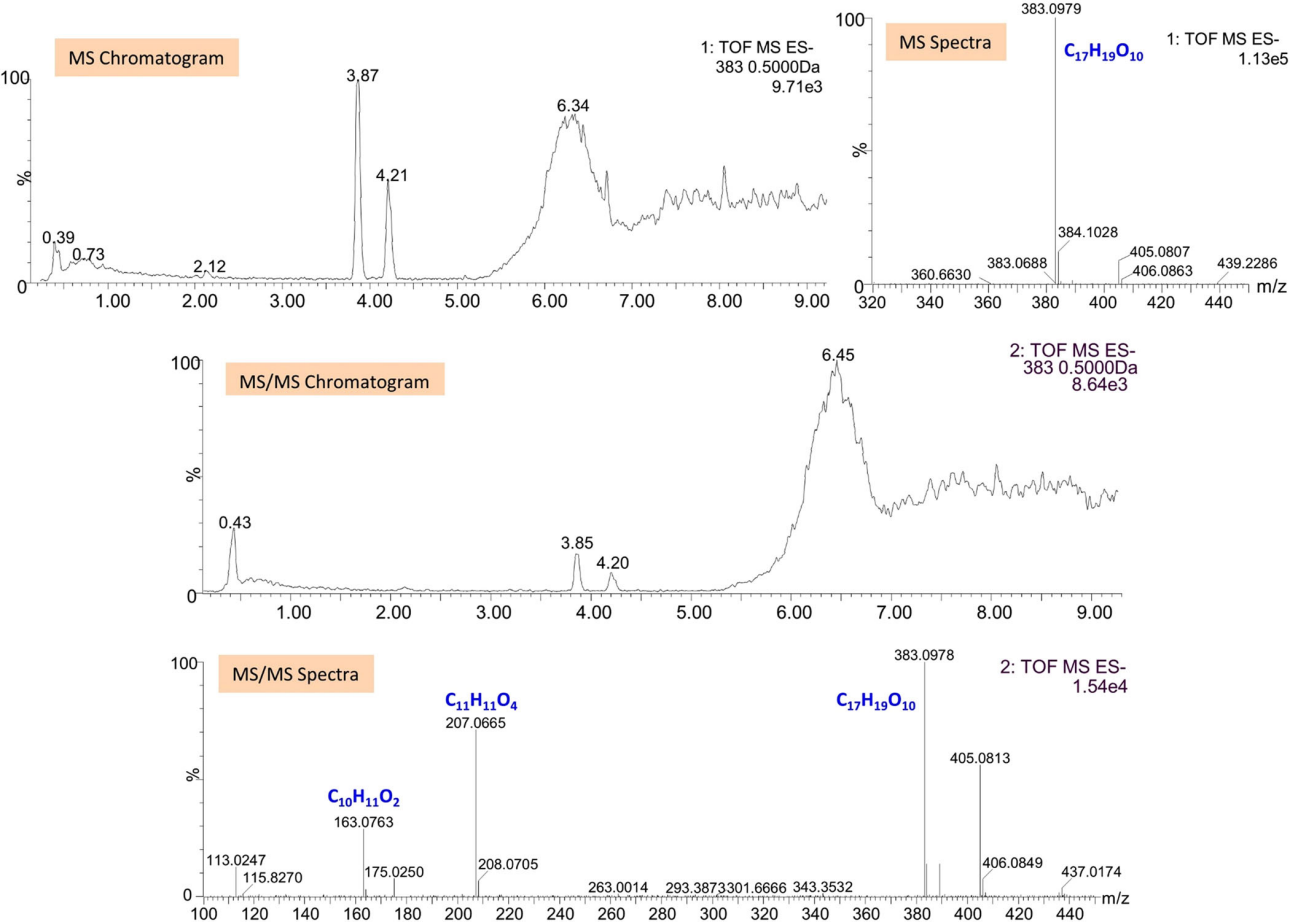
VLG1 and VLG2 LC‐HRMS chromatograms and spectra. ES, electrospray; LC‐HRMS, liquid chromatography with high‐resolution mass spectrometer; MS, mass spectrometry; TOF, time‐of‐flight; VLG, valerolactone glucuronide.

Most importantly, it is observed that VL is rapidly metabolized (*t*_½_ 8.72 min) in the S9 fraction and over time two different phase II metabolites are formed, both glucuronic adducts, on the two possible conjugation sites of the molecule. Their identity is confirmed by LC‐HRMS: valerolactone glucuronide 1 (VLG1) and 2 (VLG2) were eluted at 3.87 and 4.21 min, respectively, which corresponds to glucuronidation at different hydroxyl moieties. Chemical structures of VLG1 and VLG2 are shown in Figure 11. However, both metabolites showed the same molecular ions at *m/z* 383.0977 ([M–H]^−^, calc. 383.0978) and fragment ions at *m/z* 207.0664 ([M–H–C_6_H_8_O_6_]^−^) and 163.0752 ([M–H^−^ C_6_H_8_O_6_–CO_2_]^−^) (Figure 12), hence are not distinguishable by this technique.

**Figure 13:**
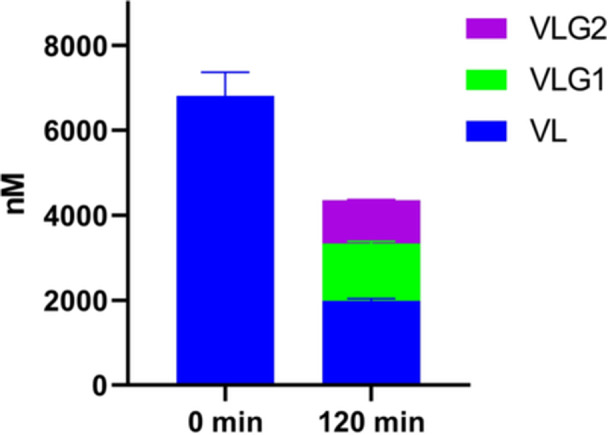
S9 liver fraction metabolism: sum of VL, VLG1, and VLG2 at the beginning of the experiment (time 0 min) and at the end of it (time 120 min). VL, 5‐(3′,4′‐dihydroxyphenyl)‐γ‐valerolactone; VLG, valerolactone glucuronide.

In the liver fraction, 70.83% of VL is metabolized and 49.06% of this is represented by the sum of VLG1 and VLG2, as shown in Figure 13. Considering the limitations due to the in vitro experimental conditions, such as the oxidation of tested compounds and protein binding, at the hepatic level VL is predominantly converted into its phase II conjugates VLG1 and VLG2, which represent the most abundant metabolites.

### 2.5 Cytochrome P (CYP) and pregnane X receptor (PXR) modulating activity

**Figure 14:**
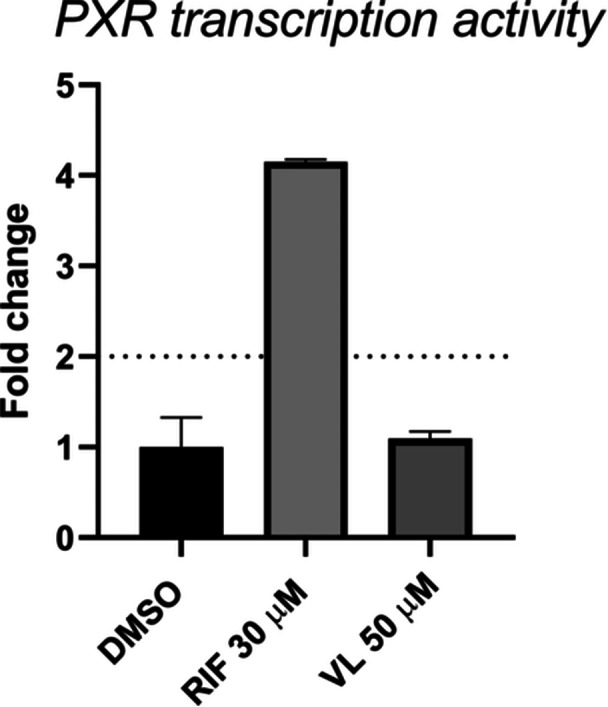
PXR modulating activity: VL shows no significant induction in PXR transcription. DMSO, dimethyl sulfoxide; PXR, pregnane X receptor; RIF, rifampicin; VL, 5‐(3′,4′‐dihydroxyphenyl)‐γ‐valerolactone.

In both LS174T and HepG2 cell lines at all concentrations tested, VL does not increase the PXR activity. RF (10 µM), a well‐known PXR activator, increased PXR activation by 2.2‐ and 3.4‐folds in the LS174T and HepG2 cells, respectively. Additionally, VL has no inhibition effect on CYP1A2 and only slightly inhibits CYP3A4 with a half‐maximal inhibitory concentration (IC_50_) value of >25 µM (Figure 14). Positive controls α‐naphthoflavone and ketoconazole IC_50_ values are comparable with previously reported data,^[^ ^25^ ^]^ hence obtained data are confirmed. These results indicate that VL is a safe metabolite with no effect on PXR activation or modulation of the expression of downstream‐targeted genes involved in drug metabolism.

Several isoenzymes from the CYPs family are involved in drug metabolization, however, CYP3A4 and CYP1A2 play a major role in the xenobiotics metabolization as they are the responsible for the metabolization of ~54% of marketed drugs.^[^ ^26^, ^27^ ^]^ CYP inhibition assays were carried out to determine whether VL could inhibit CYP3A4 and CYP1A2. The activity of CYP3A4 and CYP1A2 isoforms was not inhibited by the compound, in fact VL has no inhibition effect on CYP1A2 and slightly inhibits CYP3A4 with an IC_50_ value of >25 µM. Positive controls α‐naphthoflavone and ketoconazole IC_50_ values are comparable with previously reported data,^[^ ^25^ ^]^ hence obtained data are confirmed.

As VL showed a slight inhibition activity of CYP3A4, to predict the drug interaction potential of the compound also in terms of PXR activation, reporter gene assays were carried out to measure the transcriptional activity of PXR. Activation of PXR is known to result in increased expression of CYP3A4 which is a major CYP isoform responsible for metabolizing the majority of clinical drugs.^[^ ^3^ ^]^ In both LS174T and HepG2 cell lines at all concentrations tested of VL, PXR transcriptional activity was not activated as the fold increase is comparable to the vehicle‐treated control (<twofold) and only when a fold induction is two or more in the PXR transcription is it considered significant. RIF (10 µM), a well‐known PXR activator, increased PXR activation by 2.2‐ and 3.4‐folds in LS174T and HepG2 cells, respectively. This result shows that VL is less likely to pose a risk of interference with the pharmacokinetics of drugs which are CYP3A4 and CYP1A2 substrates.

### 2.6 Predicted ADME properties

**Figure 15:**
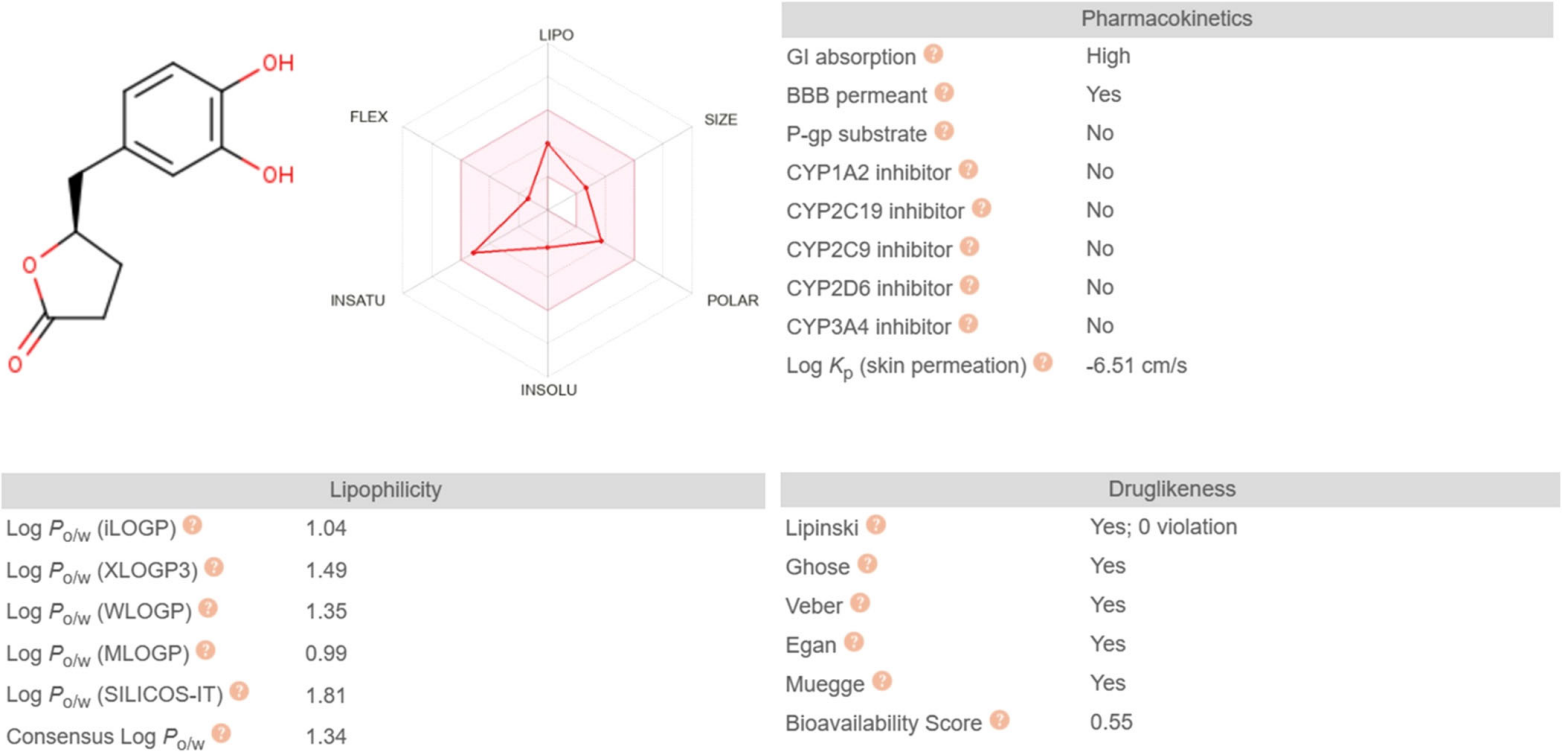
SwissADME in silico ADME properties of VL: VL shows acceptable drug‐like properties. FLEX, flexibility; INSATU, insaturations; INSOLU, insolubility; LIPO, lipophilicity; P‐gp, *P*‐glycoprotein; POLAR, polarity; VL, 5‐(3′,4′‐dihydroxyphenyl)‐γ‐valerolactone.

VL in silico ADME properties, evaluated with the online tool SwissADME, confirm the good potential of the metabolite to be used as a drug; in particular, its pharmacokinetic properties are optimal for oral administration as it is highly GI absorbable and is not a P‐gp or a cytochrome substrate. The compound also follows all the drug likeness rules with no violation of the Lipinski rule of 5. On the contrary, evaluating the bioavailability radar graph reported in Figure 15 the molecular structure has a low flexibility and size.

**Figure 16:**
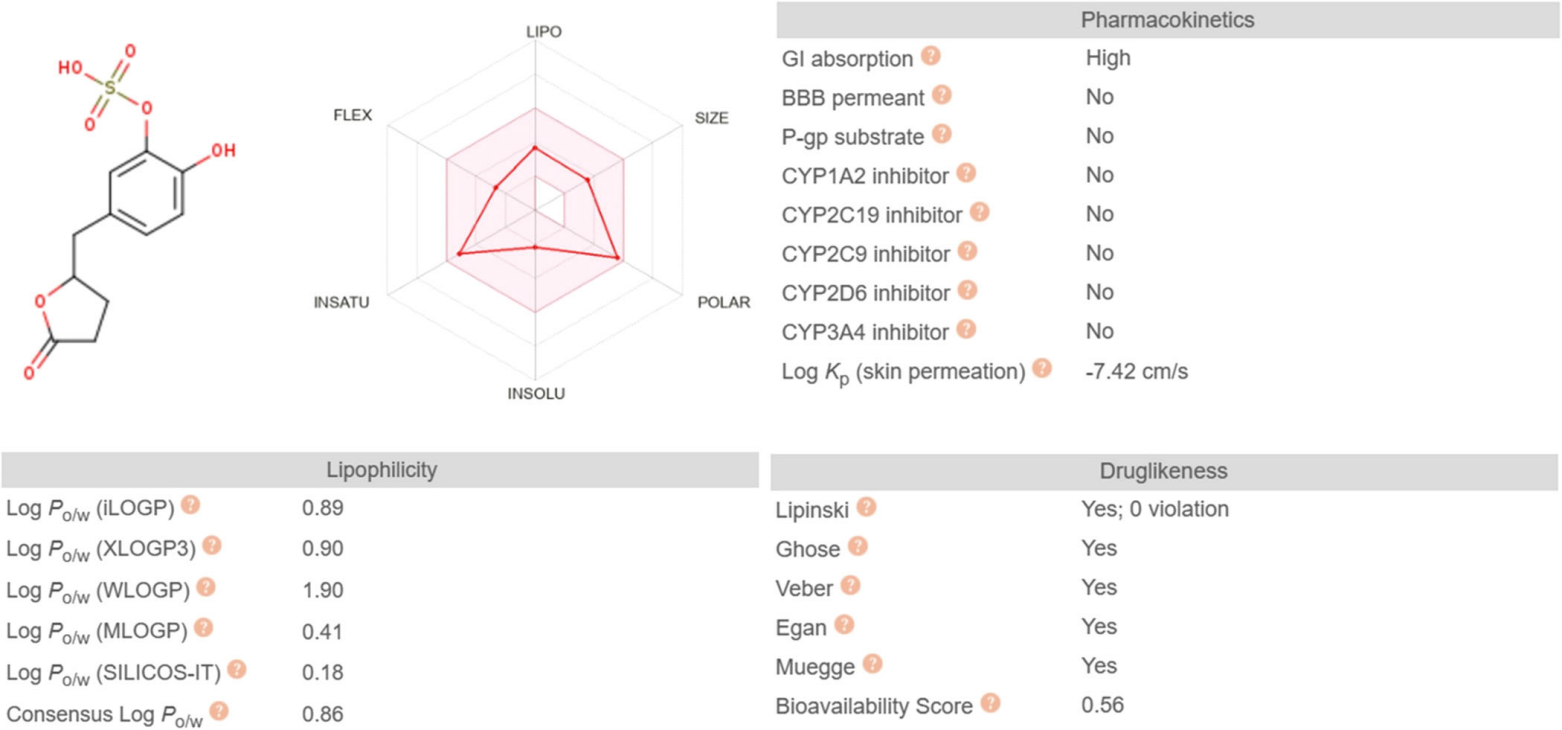
SwissADME in silico ADME properties of VLS: VLS shows acceptable drug‐like properties. FLEX, flexibility; INSATU, insaturations; INSOLU, insolubility; LIPO, lipophilicity; P‐gp, *P*‐glycoprotein; POLAR, polarity; VLS, valerolactone sulfate.

Considerations reported in Section 2.2 regarding the efflux ratio of VLS, in silico ADME properties of VLS, are evaluated, focusing attention on the lipophilicity and drug‐like properties of the metabolite. In fact, to determine whether the efflux or the absorption of VLS is predominant, its affinity to efflux transporters and lipophilicity were calculated in silico. Figure 16 shows the results obtained and, as VLS is not a substrate of P‐gp, it has optimal drug‐like properties and lipophilicity. These aforementioned properties are closely related to the rate of passive transport of the metabolite: drugs considered optimal for intestinal passive diffusion have a molecular weight lower than 200 Da and balanced hydrophilicity.^[^ ^28^ ^]^ The molecular weight of VLS is 288 g/mol, but its bioavailability score is 0.56, hence VLS can be considered an ideal and easily absorbed molecule.^[^ ^29^ ^]^ Additionally, the consensus Log *P*_o/w_ of VLS is 0.86,^[^ ^30^ ^]^ so in terms of lipophilicity it has optimal and balanced characteristics for passive diffusion and intestinal absorption.

### 2.7 Discussion

Caco2 cells are well known for their extensive phase II metabolism and efflux activity. Moreover, the formation of conjugate metabolites and their efflux are interrelated processes, as the enzymes and efflux proteins work serially. Conjugated metabolites produced by enzymes such as UGTs or SULTs (sulfotrasferares) are substrates for the apical transporters Multidrug Resistance‐associated Protein 2 (MRP2), BCRP or for basal localized transporters, like, MRP3. In this scenario, the role of efflux proteins is to prevent intracellular accumulation of phase II metabolites and this interaction has so far been studied mainly for flavonoids and toxins.^[^ ^31^ ^]^ In the present study, VL is rapidly metabolized by Caco2 phase II enzymes in its more hydrophilic conjugate VLS. At time 0 check‐point this metabolite is not present but it is already relatively quantifiable in the receiving compartment after 30 min of incubation. Hence, the rate of formation of VLS is very rapid and at the end of the experiment (120 min) VLS is present and quantifiable in the receiving compartment and it is present and quantifiable in a higher amount in the donor compartment; there VL is no longer detectable as it has been completely metabolized into its sulfate derivative. For these reasons, it was not possible to evaluate the *P*_app_ value of VL as it is completely metabolized at the tested concentration of 50 µM. On the basis of previous studies, this concentration is more than 100 times higher than the intestinal reached concentration of VL in 24/h in an in vivo pilot study after the consumption of 300 mg/day supplement dosed in PACs, but considering the limitations of the in vitro model itself and the tested cell viability, the results can be considered reliable.^[^ ^13^ ^]^ Results obtained through this in vitro model highlight how, even if the tested concentration on differentiated colonocytes is higher than the in vivo reached concentration, phase II enzymes physiologically present in the intestinal barrier are not saturated and completely transform VL into its sulfate derivative. This metabolite is a substrate of efflux transporters so it is excreted in the lumen side (A compartment), but is also absorbed and reaches the blood circulation (B compartment). In previous studies, the way the efflux transport can influence the phase II metabolite absorption has been shown. Hu et al.^[^ ^32^ ^]^ evaluated an apigenin uptake in a Caco2 cell model, the excretion rates of apigenin sulfate and glucuronide were much slower than the formation of conjugated metabolites, indicating that efflux transport may be the limiting step for apigenin conjugate elimination. Thus, this study shows that efflux transport is less efficient than the formation of metabolites, resulting in either an inhibition of phase II metabolization or in a considerable conjugate uptake. Also, the lipophilicity and drug‐like properties of VLS calculated with SwissADME confirm that the phase II metabolite of VL possesses physicochemical properties superimposable to a drug with a high rate of intestinal passive transport, confirming the hypothesis that the efflux transport is less efficient than the absorption of the conjugate.

To confirm the data obtained with the Caco2 model, the same transport study was performed using a Wt‐MDCK model. This model is commonly reported in the literature even though it is less recommended, as the cell is canine and renal. Despite this, the reproducibility of the data obtained and the enzymes and transporters expressed ensure the validity of this cell model even though it is not as reliable as the human model. In addition, Wt‐MDCK forms a differentiated monolayer in only 5 days, allowing the evaluation of the permeability of compounds on a less senescent cell model when compared with the Caco2 monolayer that requires 21 days to be differentiated.^[^ ^33^ ^]^

The efflux ratio permits the evaluation of whether any efflux transporters are involved. Drugs with an efflux ratio close to one suggest that there is an equal directional flux, and that passive diffusion is the predominant mechanism involved in the absorption. For drugs with an efflux ratio greater than 2, there is a definite contribution of efflux transporters in transport mechanisms. In this study, an efflux ratio of 2.351 for VLS in the Caco2 model and of 0.843 in the Wt‐MDCK model indicates that specific efflux transporters expressed in the Caco2 model are involved in its efflux, apparently reducing the rate of VLS absorption. In fact, despite this considerable efflux ratio, *P*_app_ in absorption direction of VLS in the Caco2 and Wt‐MDCK models (2.225 × 10^−5^ and 1.733 × 10^−5^ cm/s, respectively) is on the same scale of propranolol (1.539 × 10^−5^ cm/s), confirming once again that VLS can be considered highly absorbable in both cellular models. Since the efflux ratio of VLS was greater than 2 in the Caco2 model, we investigated the major differences in expressed efflux transporters in the Caco2 and Wt‐MDCK models. Caco2 spontaneously differentiates into enterocytes forming columnar epithelial cells with tight junctions and brush border; for these reasons Caco2 are the most relevant models to study DDI and HDI in intestinal transport. Transporters are membrane‐bound proteins and their role in cellular homeostasis is crucial, in fact they regulate the transport of nutrients, endogenous compounds, drugs and toxins. Caco2 expresses both efflux and uptake transporters; the main efflux transporters are P‐gp/MDR1 (ATP‐Binding Cassette B1 or ABCB1), MRP2 (ABCC2), MRP4 (ABCC4), and BCRP (ABCG2). The Wt‐MDCK cells were isolated from canine distal renal tissue and differentiated spontaneously into columnar epithelium forming tight junctions and are a well‐assessed model for membrane permeability evaluation in early discovery drug studies. Because of their canine origin, the main efflux transporters expressed in this cell line are canine P‐gp/Mdr1, Mrp1, Mrp2, and Mrp5.^[^ ^34^ ^]^ The main difference between the two cellular models is the expression of the efflux transporter BCRP, which is present in the Caco2 and significantly less expressed in the Wt‐MDCK model. Focusing the attention on the difference between the VLS efflux ratio and the different expressions of the BCRP transporter, it is possible to conclude that VLS is both substrate and activator of the BCRP transporter. This hypothesis is confirmed by the P‐gp inhibition assay results. In fact, when cyclosporin A (20 µM), a well‐known selective inhibitor of P‐gp, is coincubated with VL (50 and 25 µM) for 90 min, there is no significant difference in Rh‐123 intracellular accumulation compared with the control (cyclosporin A 20 µM only), hence VLS has no effect on P‐gp modulation. On the other hand, when elacridar (2 µM), a dual inhibitor of P‐gp and BCRP, is coincubated with VL (50 and 25 µM) for the same amount of time, the result is a significantly reduced accumulation of intracellular Rh‐123, hence the BCRP efflux transporter is actively modulated by the tested compound, in this study VLS, because of the conversion rate and experimental time as explained above. The results obtained are in line with previous studies reported in the literature. In fact, it is reported that natural compounds such as flavonoids are subjected to extensive phase II metabolism in Caco2 cellular models and are preferential substrates of efflux transporters, such as MRPs and BCRPs.^[^ ^35^ ^]^ Also, VLS is a BCRP substrate and activator only at a higher dosage than that reached in in vivo studies;^[^ ^13^ ^]^ hence, this effect is observed only as the experiment performed was an in vitro evaluation. In in vitro metabolization of VL in HLMs and S9 fractions, it can be observed that VL is slowly metabolized in HLM fraction and that phase I metabolism is slower (*T*_1/2_ 23.084 min) than phase II metabolism, where VL is rapidly metabolized in S9 fraction (*T*_1/2_ 8.72 min). By studying the formation of the conjugate metabolites during the reaction, it is possible to notice that as the concentration of VL decreases, the concentration of the two glucuronide conjugates increases. However, combining the in vitro absorption results, the presence of the free form of VL at the hepatic level does not seem to be possible. According to Coughtrie et al.^[^ ^36^ ^]^ the sulfation pathway is reversible, and the inversion is mainly mediated by arylsulfatase (ARS), which is a lysosomal enzyme with a higher messenger RNA expression at the hepatic level.^[^ ^37^ ^]^ Specifically, its B isoform (ARSB) mediates the hydrolysis of sulfate conjugates of phenolic compounds by restoring the OH group to its free form. The activity of this enzyme could therefore explain how after the formation and absorption of VLS it can reach the liver, where an amount can be cleaved and converted to glucuronic derivatives.^[^ ^38^ ^]^ This aspect is crucial as previous data demonstrate that VL is the active form with anti‐inflammatory and antioxidant activities:^[^ ^13^ ^]^ in vivo evidence reports the presence in the urine of sulfate and glucuronic VL conjugates, moreover, correlated data highlight the key role of the catechol moiety in VL, as it is directly involved in the Nrf2/Nf‐kB pathway. For these reasons the cleavage of the sulfate group and restoration of the catechol not only explains the formation of VLG1 and VLG2 in the liver after complete conjugation and absorption of VLS, but also the activity of VL as such. Moreover, several studies have evaluated the conjugates bioavailability and biological activity demonstrating that the crucial step is their deconjugation. Sulfatases and glucuronidases are deconjugating enzymes which convert conjugated metabolites into their unconjugated forms. Arylsulfatases and β‐glucuronidases are the most representative deconjugation enzymes and both are ubiquitously expressed, but the second is found at higher concentrations in the liver, in the small intestine, and in tissues with inflammatory cells, such as lymphocytes and/or macrophages.^[^ ^39^ ^]^ Bartholomé et al. demonstrated that activated neutrophils generate substantial quantities of β‐glucuronidases, enzymes that play a key role in deconjugating phase II metabolites circulating in the bloodstream.^[^ ^40^ ^]^ These evidences lead to support the deconjugation of VL phase II metabolites at the inflammation sites, thus contributing to its activity in the VL form. Also, a human clinical study reported by Perez et al.^[^ ^41^ ^]^ is demonstrated the importance of deconjugation: the double‐blind, placebo‐controlled, randomized trial showed that increased branchial arterial diameter is attributable to dietary quercetin, which exerts acute vasodilator effects in vivo due to the deconjugation of the metabolite quercetin 3‐glucuronide.

In vivo, the two VL metabolites 3ʹ‐OH‐PVL‐4ʹ‐sulfate and 4ʹ‐OH‐PVL‐3ʹ‐glucuronide are the main PACs metabolites detected in human plasma and urine after PACs oral intake, with 3ʹ‐OH‐PVL‐4ʹ‐sulfate being the predominant of the two. The formation of these phase II metabolites after PACs intake is well known and documented since 2000, when they were detected in a clinical trial after the intake of 960 mg of a procyanidin fraction of French maritime pine bark extract.^[^ ^42^ ^]^ In addition, in plasma, 3ʹ‐OH‐PVL‐4ʹ‐sulfate is reported to be the most abundant with a *C*_MAX_ (mean ± SD) of 368.4 ± 155.7 nmol/L, while 4ʹ‐OH‐PVL‐3ʹ‐glucuronide is found at 228.6 ± 345 nmol/L, as recently described by Di Pede et al. who reviewed the results of human ADME studies of PACs in healthy subjects.^[^ ^43^ ^]^ The ratio is also maintained in the urine with an average urinary excretion (% of ingested dose) of 11.3% ± 14.8% and 3.1% ± 3.3% for 3ʹ‐OH‐PVL‐4ʹ‐sulfate and 4ʹ‐OH‐PVL‐3ʹ‐glucuronide, respectively. Our results could be seen as being in line with these data by considering the rapid and immediate conversion of VL to 3ʹ‐OH‐PVL‐4ʹ‐sulfate (100% conversion after 30 min) that occurs in the intestine; once the VL reaches the liver it could be partially desulfated and converted into 4ʹ‐OH‐PVL‐3ʹ‐glucuronide. S9 liver fraction shows the appearance of VLG1 after 30 min, at a concentration 10 times lower than that of VL (reaching a maximum concentration at 120 min, 6 times lower than VL), meaning that this conversion occurs in a lower extent with respect to that of VL into VLS. These data are in line with the highest concentration of VLS with respect to VLG as found in vivo. Clearly, taking into account that our in vitro model has the limitation of being a nondynamic system, our hypothesis that the conversion of VL to sulfate is followed by a partial desulfation by arylsulfatase and then glucuronidation, requires an experimental confirmation that is in process in our laboratory.

VL showed negligible inhibitory effect on the catalytic activity of CYP3A4 while no inhibition of the activity of CYP1A2 was observed. Additionally, VL showed < two‐fold induction in PXR activity and for these reasons predictive results obtained using the SwissADME online tool are confirmed also by the experimental assays. Taken together, these outcomes suggest that the concomitant formation of VL metabolite with prescription medications is less likely to pose any risk of pharmacokinetic drug interactions.

## 3 LIMITATIONS OF THE PRESENT STUDY

The results shown in this work are obtained using in vitro models. Specific cell culture models have been chosen to mimic the relevant in vivo cell biology and physiology but still it is challenging to reproduce the complexity of the human body with an in vitro model. In particular, we have already discussed in the text the limitations of the Wt‐MDCK model and those regarding a nondynamic system for the study of the conversion of VL into their metabolites. Another limitation of the present study regards the stereochemistry of VL: in this study and in our previous one reported by Baron et al.,^[^ ^13^ ^]^ we used a mixture (*R*/*S*) due to the complexity and cost of an enantioselective synthesis, but not allowing to fully reflect the in vivo situation. Moreover, in our studies, it was not possible to evaluate whether an enantioselective metabolism of VL and derivatives can occur since the *R*‐ or *S*‐enantiomers coelute when analyzed in reverse‐phase liquid chromatography.^[^ ^15^ ^]^ However, this is an important aspect that will be the subject of future studies.

## 4 CONCLUSION

In conclusion, VL is the main metabolite after intake of PACs and flavan‐3‐ols‐rich supplements and is formed by intestinal microbiota metabolism. It is the main active metabolite as it is widely absorbed as a sulfate conjugate in the intestine and reaches the systemic circulation. In addition, it can be converted into its free form by an arylsulfatase enzyme. In the liver, VL can be further metabolized into its glucuronide adducts, which represent the predominant hepatic metabolites of the metabolite, and VL neither induces nor blocks major cytochromes and gene receptors that can alter their expression, nor efflux systems causing no HDI. Furthermore, VL can represent a scaffold for the LEAD optimization process as it is a molecule with safe pre‐ADMET properties, promising in vivo activities with encountered evidence and possesses optimal chemical parameters to represent the starting point for SAR studies and the synthesis of new drugs with a potent antioxidant and anti‐inflammatory capacity.

## 5 EXPERIMENTAL

### 5.1 Biochemicals and reagents

VL (purity grade >99.5%, see the Supporting Information) was synthesized following the procedure reported by Artasensi et al.^[^ ^44^ ^]^ and Baron et al.^[^ ^45^ ^]^ DMEM‐F12, Dulbecco's Modified Eagle Medium (DMEM), 2‐[4‐(2‐hydroxyethyl)piperazin‐1‐yl]ethanesulfonic acid, trypsin ethylenediaminetetraacetic acid, penicillin–streptomycin solution, and sodium pyruvate were purchased from GIBCO BRL, Invitrogen Corp. Fetal bovine serum (FBS) was purchased from Hyclone Lab Inc. Propranolol, paclitaxel, testosterone, 7‐hydroxycoumarin, RIF, camptothecin, CsA, elacridar, 3‐[4,5‐dimethylthiazol‐2‐yl]‐2,5 diphenyl tetrazolium bromide (MTT), DMSO, phosphate‐buffered saline (PBS), Hank's buffer, rhodamine‐123, G‐6‐PDH, glucose‐6‐phosphate, nicotinamide adenine dinucleotide phosphate (NADP^+^), and uridine diphosphate glucuronic acid (UDPGA) were obtained from Sigma Chem. Co. PXR reporter assay system was procured from Indigo Biosciences. All other reagents and solvents were of analytical grade and procured from authentic sources. HLMs and S9 fractions (pooled mixed sex) were from In Vitro Technologies Inc. CYP induction assay kits (P450‐GloTM) were obtained from Promega Corporation.

### 5.2 Cell culture

Caco2 (human colorectal adenocarcinoma), Wt‐MDCK (wild type Madin‐Darby Canine Kidney), HepG2 (human hepatocellular carcinoma), and LS174T (human colon adenocarcinoma) cell lines were obtained from the American Type Culture Collection. The HepG2 and LS174T cells were maintained and routinely cultured in DMEM/F12 medium. Wt‐MDCK and Caco‐2 cell lines were cultured and maintained in a high‐glucose DMEM/F12 medium supplemented with 1% nonessential amino acids, and 1% l‐glutamine. Additionally, all media were supplemented with 10% heat inactivated FBS, 2.4 g/L sodium bicarbonate, 100 μg/mL streptomycin, and 100 U/mL penicillin. Cells were grown at 37°C in an environment of 5% CO_2_ and 98% relative humidity. Stock solutions of the tested compounds and controls were diluted in phenol red and serum‐free DMEM/F12 media to the desired concentrations, and the DMSO concentration did not exceed 0.3% (v/v) during treatments.^[^ ^46^, ^47^ ^]^

### 5.3 Cell viability assay

The effect of test samples on cell viability was measured using an MTT assay. Briefly, exponentially growing 2 × 10^4^ cells/200 μL were seeded in 96‐well plates and allowed to grow until reaching their differentiation state. Cells were treated with different concentrations of VL (50–1.6 µM) and positive control camptothecin (30–1 μM) serially diluted in serum‐free medium. After incubation, 10 μL of MTT dye (5 mg/mL stock in PBS) was added to each well, and plates were additionally incubated for 4 h. Further, media were aspirated, and cells were washed with 100 μL of phosphate buffer saline. The formazan blue crystals formed by viable cells were dissolved in 150 μL of DMSO, and the absorbance was measured at 580 nm on a Bio‐Tek, Synergy HT Multi‐Mode, plate reader. The % decrease in viability of sample‐treated cells was calculated compared with vehicle‐treated cells.^[^ ^46^ ^]^

### 5.4 In vitro transport study across Caco2 and Wt‐MDCK monolayers

Caco2 cells between passage numbers 42–50 and MDCK cells between passage numbers 21–26 were used for transport studies; the assay was conducted as reported by Volpe^[^ ^33^ ^]^ with slight modifications. Transport experiments were performed in 12‐well Transwell plates. Caco2 cells were seeded at a density of 60,000 cells/cm^2^ and grown for 21 days, with change of media every 3 days, while MDCK cells were seeded at a density of 70,000 cells/cm^2^ and grown for 5 days, changing the medium daily. The physiological and morphological formation of a confluent monolayer was evaluated by measuring the TEER value: Caco2 monolayers became confluent 1 week after seeding with TEER values greater than 450 Ω/cm^2^, while MDCK monolayers became confluent 24 h after seeding with TEER values greater than 90 Ω/cm^2^. Phenol red and serum‐free DMEM/F12 medium was used as transport buffer. For bidirectional transport, VL (50 μM) and control compound (propranolol and paclitaxel, 10 μM) were added to the apical side to determine apical to basolateral transport (*A*–*B*; absorptive direction) and to the basal side to determine basolateral to apical transport (*B*–*A*; secretory direction). The volume of the apical and basolateral chambers was 0.5 and 1.5 mL, respectively. Aliquots of 120 μL were taken from the basolateral (for *A*–*B* transport) or the apical (for *B*–*A* transport) chamber at 30, 60, 90, and 120 min. An equal volume was replaced with a transport buffer at every time point. At the beginning and at the end of the experiment, an aliquot was also taken from the apical or basolateral chamber for analysis of the drug. To determine the integrity of monolayers, TEER values were measured before and after the experiment. In addition, at the end of the experiment cells were collected and lysed to evaluate the compound intake. Briefly, cells were washed twice with cold PBS and 200 µL of cold MeOH was added to detach and lysate the cells; subsequently, cells were collected into lo‐bind Eppendorf and sonicated in ice for 10 min, then centrifuged for 15 min at 4°C at 14,000 rpm (Eppendorf Centrifuge 5430 R). The supernatant was collected and used for further analysis.

The apparent permeability *P*_app_ (cm/s) of compounds was calculated from the following equation (Equation 1), while *P*_app_ (cm/s) of the metabolites was calculated from the equation (Equation 2):

(1) $$P_{\mathrm{app}}=\left(\frac{\mathrm{dQ}}{\mathrm{dt}}\right)\times \frac{1}{C}\times \frac{1}{A}.$$

Equation (1) for calculating *P*_app_ (cm/s) of tested reference compounds.

(2) $$P_{\mathrm{app}}=\frac{\mathrm{Metabolic}\;\mathrm{cleareance}}{A}.$$

Equation (2) for calculating *P*_app_ (cm/s) of compound metabolites.

with

$$\mathrm{Metabolic}\;\mathrm{cleareance}=\frac{\mathrm{Rate}\;\mathrm{of}\;\mathrm{metabolism}}{C}$$

and where *dQ*/*dt* is the rate of transport, *C* is the initial concentration in the donor compartment, and *A* is the surface area of the filter. To quantify the rate of transport, cumulative amounts of test compounds were plotted against time (min).

Efflux ratio was calculated using the following equation (Equation 3)^[^ ^48^ ^]^:

(3) $$\mathrm{Efflux}\;\mathrm{ratio}=\frac{P_{\mathrm{app}}(B-A)}{P_{\mathrm{app}}(A-B)}.$$

Equation (3) for calculating efflux ratio.

### 5.5 Metabolic stability assay with human S9 liver fraction and HLM

HLMs and S9 fractions were used to determine, respectively, phases I and II metabolism of VL. A mixture of the activating cofactors (NADP^+^ and UDPGA) and enzymes (G_6_PO_4_ and G_6_PDH) were used to stimulate the metabolism; in the microsome assay UDPGA was not needed. The final concentrations of cofactors NADPH and UDPGA were 1 mM each, while for the enzymes G_6_PO_4_ and G_6_PDH were 10 mM and 2 U/mL, respectively. The phosphate buffer was prepared as a 100 mM solution, pH 7.4, containing 10 mM magnesium chloride in deionized water. Testosterone and 7‐hydroxycoumarin were used as positive controls for phases I and II metabolism, and stock reference solutions of controls and VL were prepared, respectively, at 5 mM and 10 mM concentration in DMSO, and then diluted to a final test concentration of 10 µM. S9 fraction or microsome, final concentration 1 mg/mL, were preincubated with a test compound for 5 min at 37°C in the phosphate buffer, pH 7.4, and then the reactions were initiated by adding the cofactor mixture. A negative control sample was prepared replacing the reaction enzymes with water in the presence of a reaction mixture and test compound VL. At time points 0, 10, 20, 30, 45, 60, 90, and 120 min, 100 µL aliquots of the sample mixture were removed and quenched by the addition of two volumes of ice‐cold 50:50 ACN:MeOH containing the internal standard (cinnamic acid‐d_6_, 300 ng/mL final concentration). The quenched samples were then stored at −80°C overnight and defrosted before analysis, sonicated for 10 min, and centrifuged at 14,000 rpm for 15 min at 4°C to sediment the precipitated proteins before injection onto ultra performance liquid chromatography‐mass/mass (UPLC‐MS/MS) for analysis. *T*_½_ was calculated using the interpolating point method using the nonlinear fit option of GraphPad Prism 8, while intrinsic clearance *CL'int* (mL/min/kg) was calculated as written in Equation (4) and as reported previously:^[^ ^23^ ^]^

(4) $$CL^{\prime }int=\frac{0.693\times \mathrm{Dose}}{T_{½}}.$$

Equation (4) for calculating intrinsic clearance *CL'int* (mL/min/kg).

### 5.6 Analytical method

The LC‐MS/MS analyses were carried out on a Waters Acquity UPLC I‐class system (Waters Corp.) coupled with a Xevo TQ‐S triple quadrupole MS detector using a Waters UPLC BEH C18 column (2.1 mm × 50 mm inner diameter, 1.7 µm). The instrument was controlled by Waters MassLynx 4.1 software. The column and sample temperatures were maintained at 40°C and 10°C, respectively. The mobile phase consisted of water containing 0.1% formic acid (A) and acetonitrile with 0.1% formic acid (B) at a flow rate of 0.3 mL/min in the following gradient elution: 0–4.5 min, 2%–12% B; 4.5–6.5 min, 12%–95% B; 6.5–7.0 min, 95%–100% B. The analysis was followed by a 3‐min washing procedure with 100% B and a re‐equilibration period of 3.5 min with the initial condition. The injection volume was 2 µL. The electrospray ionization (ESI) MS/MS parameters were set as follows: capillary voltage, 3.8 kV; source temperature, 150°C; desolvation temperature, 300°C; desolvation gas flow, 800 L/h; and cone gas flow, 150 L/h. Nitrogen was used as the desolvation and cone gas. Argon (99.99% purity) was introduced as a collision gas into the collision cell at a flow rate of 0.15 mL/min. The effluent was introduced into the TQ‐S mass spectrometer both in positive ion mode (ESI+) and negative ion mode (ESI−) for quantification of the analytes. Detection was obtained by MRM mode. The quantification of VL (retention time, 4.42 min, lower limit of quantification [LLoQ] 20 nM) and its sulfate (retention time, 4.08 min) and glucuronide (retention time, 3.87 and 4.21 min) metabolites were acquired with transitions of key product ions at *m/z* 207.1 → 121.8 (dwell time 4 ms, cone voltage 56 V, and collision energy 16 eV). For the quantification of propranolol (retention time, 5.81 min, LLoQ 50 nM) and paclitaxel (retention time, 6.43 min, LLoQ 10 nM), MRM ions transitions were at *m/z* 260.1 → 71.1 (dwell time 4 ms, cone voltage 4 V, and collision energy 20 eV) and *m/z* 854.2 → 121.9 (dwell time 4 ms, cone voltage 24 V, and collision energy 32 eV), respectively. For the quantification of 7‐hydroxy coumarin (retention time, 5.02 min, LLoQ 50 nM) and testosterone (retention time, 6.26 min, LLoQ 0.1 nM), MRM ions transitions were at *m/z* 162.9 → 107.1 (dwell time 3 ms, cone voltage 8 V, and collision energy 20 eV) and *m/z* 289.1 → 97.0 (dwell time 3 ms, cone voltage 8 V, and collision energy 22 eV), respectively. Cinnamic acid‐d6 (retention time, 5.93 min) has been used as the internal standard at a final concentration of 200 ng/mL, and MRM ions transition was at *m/z* 155.1 → 135.6 (dwell time 3 ms, cone voltage 28 V, and collision energy 10 eV).

VL metabolites, named VLS and valerolactone glucuronides (VLG1/VLG2), were determined on a Waters Acquity UPLC system coupled with a Xevo G2‐S quadrupole time‐of‐flight MS detector (Waters Corp.). ESI MS was operated in negative ion mode. The LC method was the same as the TQ‐S method and applied for the analysis with a Waters UPLC BEH C18 column.^[^ ^25^ ^]^

### 5.7 Rhodamine‐123 uptake assay for P‐gp and BCRP modulation

P‐gp modulation was determined in differentiated Caco2 cells by Rh‐123 uptake assay.^[^ ^49^ ^]^ In brief, cells in the logarithmic growth phase with 70%–80% confluency were harvested and seeded at a density of 2 × 10^4^/well of a 96‐well plate. Cells were allowed to grow for 21 days in a CO_2_ incubator changing the medium every other day.

#### 5.7.1 Inhibition

To evaluate P‐gp inhibition on day 21, the medium was removed and 200 μL Hank's salt solution was added, the plate was then incubated for 40 min. Further, Hank's salt solution was aspirated and 190 μL of phenol and serum‐free DMEM/F12 medium containing 10 mM of Rh‐123 was added, then 10 μL of VL (50–1.5 μM) and positive controls (CsA 20–0.6 μM; elacrydar 2–0.06 μM) were added and cells incubated for 90 min. After treatment cells were washed three times with ice‐cold PBS, and 200 μL of lysis buffer (0.1% Triton X 100 and 0.2 N NaOH) was added. The plate was kept on a shaker for 2 h at room temperature, and 100 μL of lysate was used to measure the Rh‐123 fluorescence at 485/529 nm. Further, 10 μL of lysate was taken in a separate 96‐well plate, and 190 μL Bradford reagent was added. The plate was shaken for 5–10 min, and absorbance was measured at 595 nm. Each sample was normalized by dividing the fluorescence of each sample by the total proteins present in the lysate.^[^ ^46^ ^]^

#### 5.7.2 Induction

To evaluate the P‐gp induction, on day 19 cells were washed as described above and treated for 48 h with VL (50–1.5 μM) and positive control RIF (10–0.3 μM) diluted in phenol and serum‐free DMEM/F12 medium; diluted compounds were changed every 24 h. After treatment, cells were washed with Hank's buffer and 200 μL of phenol, and serum‐free DMEM/F12 medium containing 10 mM of Rh‐123 was added and cells were incubated for 90 min. The final steps were as described previously.

### 5.8 Reporter gene assay for PXR activation

The PXR activation potential of VL was determined in transiently transfected HepG2 and LS174T cells as described earlier.^[^ ^50^ ^]^ In brief, 24‐h‐old cells having 70%−80% confluency were trypsinized and transfected with pSG5‐hPXR (25 μg) and PCR‐5 (25 μg) plasmid DNA by electroporation at 180 V (1 pulse for 70 ms). Cells were incubated at room temperature for 8 min and floating dead cells were carefully removed. Cells were seeded in 96‐well plates at a density of 50,000 cells/well. After 24 h, when cells retain >90% confluency, the tested sample VL (1.5–50 μM) and positive control (RIF: 10 μM) were added. Following 24 h of incubation, the culture medium was aspirated and 40 μL of luciferase reagent (Promega Corporation) was added to each well. Luminescence was measured using a Spectramax M5 plate reader (Molecular Devices) and the fold increase in luciferase activity among sample‐treated cells was calculated in comparison with the vehicle‐treated (DMSO) cells.

### 5.9 CYP inhibition assay

The inhibitory potential of VL toward the catalytic activity of CYP3A4 and CYP1A2 was investigated through CYP inhibition assays using high‐throughput CYP450 screening kits. Stock solutions of VL (10 mM in DMSO) and specific positive controls for each CYP isoform were serially diluted in methanol and reactions were performed in 96‐well plates according to the supplier's instructions. Briefly, 40 μL (×2.5) of test compound and 50 μL master premix were added to 96‐well plates. Plates were incubated for 10 min at room temperature to allow for sufficient interaction of the test compound and enzyme. Following incubation, 10 μL (×10) of enzyme‐specific substrates were added and plates were vortex‐mixed for 30 s. Further, fluorescence was measured at the specified excitation and emission wavelengths for each substrate as recommended per individual kit protocol. Ketoconazole (0.004, 0.01, 0.03, 0.11, 0.33, and 1 μM) and α‐naphthoflavone (0.004, 0.01, 0.03, 0.11, 0.33, and 1 μM) were used as positive controls for CYP3A4 and CYP1A2, respectively. IC_50_ values were obtained from concentration‐response curves generated by plotting the percentage of inhibition versus concentration.^[^ ^46^ ^]^

### 5.10 In silico ADME prediction

To evaluate the in silico ADME properties of VL, the online ADME prediction tool SwissADME was used to predict its drug‐like and the pharmacokinetic properties, in particular the lipophilicity (Log *P*_o/w_), pharmacokinetics (gastrointestinal absorption, blood brain barrier permanent, P‐gp substrate, CYP 1A2/2C19/2C9/2D6/3A4 inhibition) and drug‐like properties (Lipinski's rule of 5).^[^ ^30^ ^]^

## CONFLICT OF INTEREST STATEMENT

The authors declare the following financial interests/personal relationships which may be considered as potential competing interests: Paolo Morazzoni has a consultancy agreement with Distillerie Bonollo Umberto S.p.A. in the field of research and development of new products based on the supply chain of Vitis vinifera L.

## Supporting information

Supporting information.

## Data Availability

The data that support the findings of this study are available from the corresponding author upon reasonable request.
