# Supplementary material for: Pre‐ADMET studies of 5‐(3′,4′‐dihydroxyphenyl)‐γ‐valerolactone, the bioactive intestinal metabolite of proanthocyanidins
Source: Arch Pharm (Weinheim). 2024 Nov 11;358(1):e2400575. doi: 10.1002/ardp.202400575 (PMC11726150; doi:10.1002/ardp.202400575)
Supplement: Supplementary file 1 — Supporting information. [file ARDP-358-e2400575-s001.docx]

**SUPPLEMENTARY INFORMATION**

The purity of VL was determined by using HPLC coupled with a HRMS detector with a full MS method as reported by Della Vedova et al.^1^. In more detail, VL a stock solution was prepared in 100% CH_3_OH and diluted for analysis to a final concentration of 50 µg/mL using H_2_O/HCOOH, 100/0.1, % v/v. A volume of 10 µL of the sample was analyzed in triplicate. For purity calculation, the blank signal was subtracted, and peak area of VL (RT 9.37 minutes) integrated from the TIC. Purity was calculated as percentage of the compound area on the total area of peaks detected and it is 98.62 % ± 0.88 (calculated mean and standard deviation). **Figure 1si** shows in panel *A* the chromatographic elution of VL, and in panel *B* VL isotopic patter.


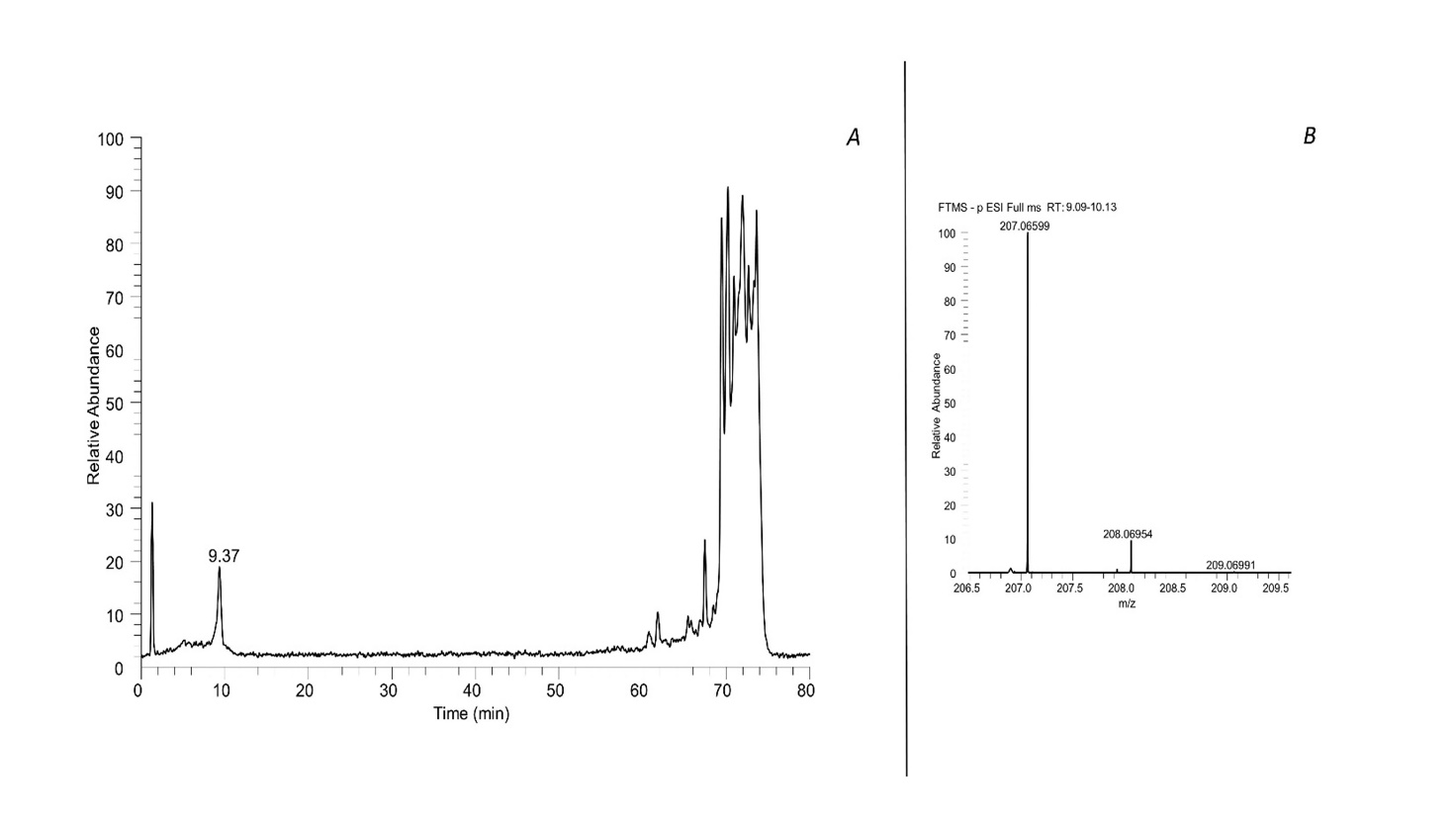


**Figure 1 si** – VL chromatogram (panel A) and VL isotopic pattern (panel B).

The synthesis of VL was accomplished following the procedure already reported in the literature by Artasensi et al.^2^ and by Baron et al.^3^. ^1^H NMR spectra were recorded using a FT-spectrometer operating at 300 MHz. Chemical shifts are reported in ppm relative to residual solvent as internal standard. Signal multiplicity is designed according to the following abbreviations: dd = doublet of doublets, m = multiplet. In **Figure 2 si** the ^1^H NMR spectra of VL is reported.

^1^H NMR (300 MHz, CD_3_OD) δ = 6.70 – 6.68 (m, 2H), 6.56 (dd, J = 7.9, 2.2 Hz, 1H), 4.74 – 4.70 (m, 1H), 2.91-2.75 (m, 2H), 2.54 – 2.45 (m, 1H), 2.42 – 2.19 (m, 2H), 1.99 – 1.92 (m, 1H).


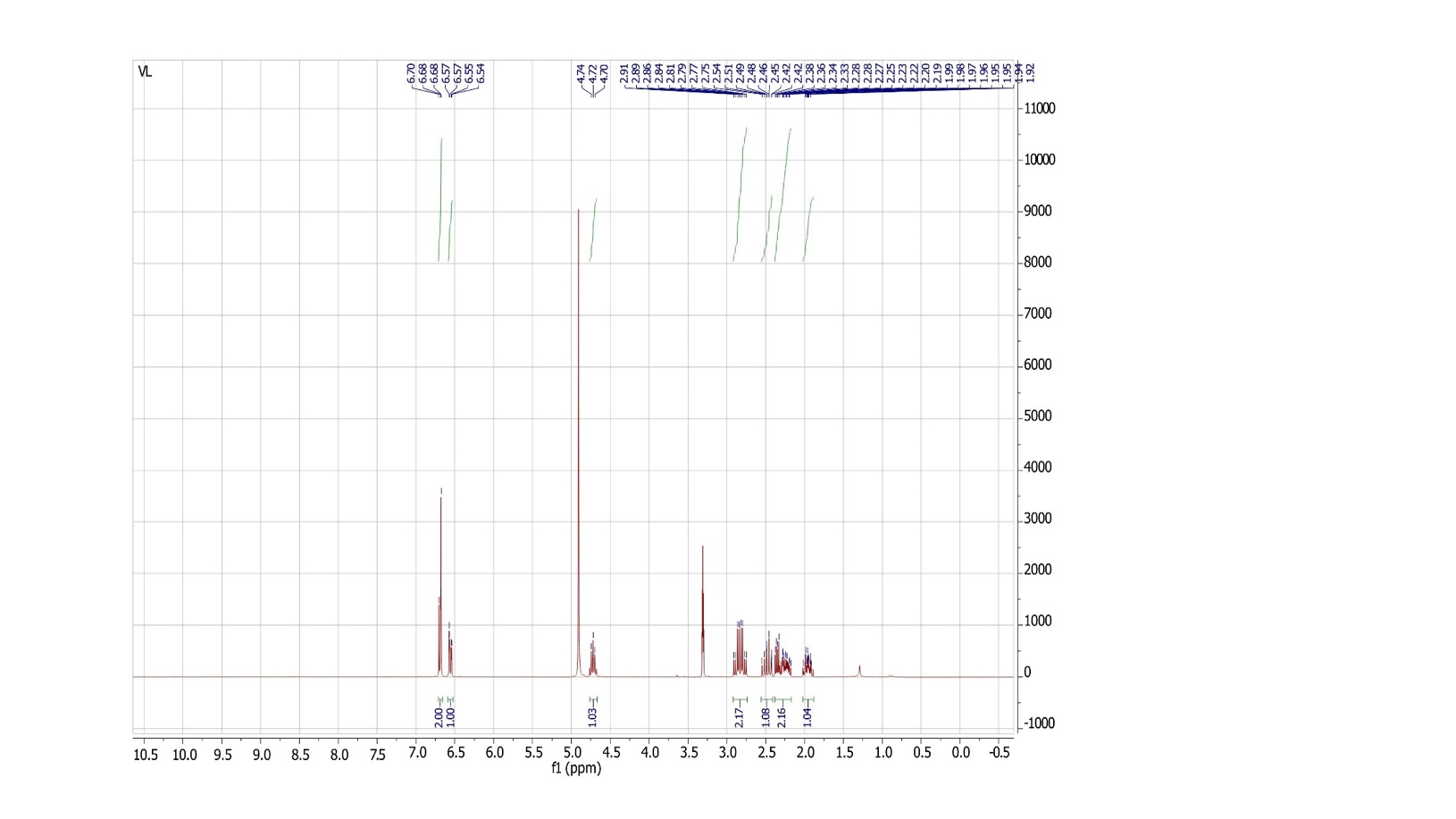


**Figure 2 si** – VL ^1^H NMR spectra.

**References**

[1] L. Della Vedova, F. Gado, T. A. Vieira, N. A. Grandini, T. L. N. Palácio, J. S. Siqueira, M. Carini, E. Bombardelli, C. R. Correa, G. Aldini, G. Baron, *Molecules* **2023,** 28, 2964. DOI: 10.3390/molecules28072964

[2] A. Artasensi, G. Baron, G. Vistoli, G. Aldini, L. Fumagalli, *Molbank* **2021**, 1, M1193. DOI: 10.3390/M1193.

[3] G. Baron, A. Altomare, L. Regazzoni, L. Fumagalli, A. Artasensi, E. Borghi, E. Ottaviano, C. Del Bo, P. Riso, P. Allegrini, G. Petrangolini, P. Morazzoni, A. Riva, L. Arnoldi, M. Carini, G. Aldini, *Biochem Pharmacol*. **2020**, 173, 113726. DOI: 10.1016/j.bcp.2019.113726. Epub 2019 Nov 26. PMID: 31778647.
